# Inflammatory Mediators and Gut Microbial Toxins Drive Colon Tumorigenesis by IL-23 Dependent Mechanism

**DOI:** 10.3390/cancers13205159

**Published:** 2021-10-14

**Authors:** Janani Panneerselvam, Venkateshwar Madka, Rajani Rai, Katherine T. Morris, Courtney W. Houchen, Parthasarathy Chandrakesan, Chinthalapally V. Rao

**Affiliations:** 1 Center for Cancer Prevention and Drug Development, The University of Oklahoma Health Sciences Center, Oklahoma City, OK 73104, USA; Janani.p1@gmail.com (J.P.); Venkateshwar-Madka@ouhsc.edu (V.M.); 2 Stephenson Cancer Center, The University of Oklahoma Health Sciences Center, Oklahoma City, OK 73104, USA; Rajani-Rai@ouhsc.edu (R.R.); Courtney-Houchen@ouhsc.edu (C.W.H.); parthasarathy-chandrakesan@ouhsc.edu (P.C.); 3 Department of Medicine, The University of Oklahoma Health Sciences Center, Oklahoma City, OK 73104, USA; 4 Department of Surgery, University of Oklahoma Health Sciences Center, Oklahoma City, OK 73104, USA; katherine-morris@ouhsc.edu; 5 VA Medical Center, Oklahoma City, OK 73104, USA

**Keywords:** colon cancer, IL-23, obesity, inflammation, innate immunity

## Abstract

**Simple Summary:**

Western-style diet, rich in high fat, is the major cause of obesity and enhanced risk of colon cancer in the USA and worldwide. The inflammatory molecules are a well-established link between obesity and the modulation of colon tumorigenesis. In particular, IL-23 plays an important role in the impact of a western-style diet on obesity, the gut microbiome, and colon tumorigenesis. However, the underlying mechanism of IL-23 production for colon tumor progression and whether IL-23 can be a potential target is not clear. Our findings signify the role of pro-tumorigenic innate immune cells, including dendritic cells and macrophages in IL-23 production by bacterial toxins and eicosanoids. IL-23 knockdown in the tumorigenic dendritic cells and macrophages inhibited the colon tumor cell and organoids growth. Taken together, targeting IL-23 may be a promising option for the prevention and treatment of high-fat/obesity-associated colon cancer in clinical trials.

**Abstract:**

Obesity-associated chronic inflammation predisposes colon cancer risk development. Interleukin-23 (IL-23) is a potential inflammatory mediator linking obesity to chronic colonic inflammation, altered gut microbiome, and colon carcinogenesis. We aimed to elucidate the role of pro-inflammatory eicosanoids and gut bacterial toxins in priming dendritic cells and macrophages for IL-23 secretion to promote colon tumor progression. To investigate the association of IL-23 with obesity and colon tumorigenesis, we utilized TCGA data set and colonic tumors from humans and preclinical models. To understand IL-23 production by inflammatory mediators and gut microbial toxins, we performed several in vitro mechanistic studies to mimic the tumor microenvironment. Colonic tumors were utilized to perform the ex vivo experiments. Our findings showed that IL-23 is elevated in obese individuals, colonic tumors and correlated with reduced disease-free survival. In vitro studies showed that IL-23 treatment increased the colon tumor cell self-renewal, migration, and invasion while disrupting epithelial barrier permeability. Co-culture experiments of educated dendritic cells/macrophages with colon cancer cells significantly increased the tumor aggression by increasing the secretory levels of IL-23, and these observations are further supported by ex vivo rat colonic tumor organotypic experiments. Our results demonstrate gut microbe toxins and eicosanoids facilitate IL-23 production, which plays an important role in obesity-associated colonic tumor progression. This newly identified nexus represents a potential target for the prevention and treatment of obesity-associated colon cancer.

## 1. Introduction

Colorectal cancer (CRC) remains a major public health issue. CRC, a highly preventable disease, continues to remain the second most lethal cancer in the US with an increasing trend globally [1]. Several epidemiological and experimental studies have shown that a western-style diet (WSD) rich in calories and saturated fat poses a risk for obesity and CRC [2]. Approximately, 30% of the US population is estimated to be overweight or obese (≥30 BMI) [3]. Further, obese patients with colon cancer exhibit chemotherapy resistance, higher rates of recurrence, and poor survival rates [4]. Preclinical studies have demonstrated that WSD rich in mixed lipids increased the colonic epithelial cell proliferation, early onset of colonic aberrant crypt foci (ACF), and colon tumor formation with a decreased apoptosis in the azoxymethane (AOM) induced preclinical models. [5,6].

Obesity leads to a low-grade chronic inflammatory state and is associated with the increased circulatory levels of pro-inflammatory mediators such as IL-23, IL-17, IL-6, IL-8, MCP-1, TNF-α, and induction of NF-κB and COX-2/PGE_2_ signaling [7]. Reports have shown that the increased level of inflammatory mediators plays critical role in the initiation and progression of colon cancer and has the potential to promote epithelial-mesenchymal transition and metastasis [8]. Furthermore, obesity-associated inflammation mediates the recruitment of innate immune cells such as macrophages, neutrophils, and dendritic cells leads to the secretion of reactive oxygen species and inflammatory mediators [8,9].

Obese individuals have been shown to possess increased gut proportions of *Firmicutes* and decreased proportions of *Bacteroidetes* with overall lower microbial genetic diversity with higher inflammatory mediators [10]. Pre-clinical studies have also shown an aberrant microbiota in genetic (Ob/Ob mice deficient in leptin production) or diet-induced (Zucker fa/fa rat) obesity animal models [11], suggesting the role of obesity in the dysbiosis of the gut microbiota and risk of colon cancer. It has been reported that commensal bacterial products such as lipopolysaccharide (LPS) and lipoteichoic acid (LTA) engage TLRs on tumor-infiltrating myeloid cells and activate MyD88 mediated production of pro-inflammatory molecules, leading to tumorigenesis [12].

IL-23 is a pro-inflammatory cytokine that belongs to an IL-12 cytokine family consisting of heterodimeric p40 and p19 subunits that act as a crucial regulator to drive a pathway that leads to the generation of IL-17–producing CD4 T cells. IL-23 is highly expressed in a broad spectrum of cancers, including colon cancer [13], and has emerged as a new player in the promotion of tumor growth and development through suppression of tumor infiltration of CD8+ T cells and the advancement of tumor angiogenesis and metastases [8,14]. Moreover, anti-IL-23 monoclonal antibody acts synergistically with targeted therapies or IL-2 to suppress tumor growth and metastases, supporting the tumor-promoting activity of IL-23 [15].

Collectively, the most common link between obesity, inflammation, and microbiota dysbiosis mediated colon cancer development and progression through the aberrant activation of innate immunity and associated pro-inflammatory molecules is predominantly IL-23. However, the underlying mechanism of obesity-associated inflammatory mediators and dysbiosis-mediated activation of innate immunity and associated IL-23 secretion for colon tumor progression require more understanding. Here, we demonstrated that WSD-associated factors such as arachidonic acid (AA), Prostaglandin E2 (PGE_2_), and bacterial toxins LTA and LPS activate pro-inflammatory macrophage and dendritic cell phenotypes to secrete IL-23 for colon tumor progression and also explored an anti-IL-23 approach for prevention and treatment of colon cancer.

## 2. Materials and Methods

### 2.1. Experimental Animals

Male and female F344 rats were housed under controlled conditions and studies were performed with approval from the Institutional Animal Care and Use Committee (IACUC) of the University of Oklahoma Health Sciences Center (OUHSC). Rats were assigned to experimental groups using simple randomization. The sample size was based on estimations by power analysis with a level of significance of 0.05 and a power of 0.9. Rats were euthanized by following IACUC approved standard CO_2_ inhalation procedure. Colonic tumors (carcinogen-induced CRC) and matched mucosa from F344 rats (RRID: RGD_1547866) were collected at termination as described earlier [16]. Samples were used for protein expression studies.

### 2.2. Human Samples

De-identified human colonic tumors were generously provided by Kathrine Morris. Patients were enrolled with informed consent, under a protocol that was reviewed and approved by the Institutional Review Boards (IRB #7565) of OUHSC. Following informed consent, a portion of resected tumor samples was collected and blinded for protein expression analysis.

### 2.3. TCGA Colorectal Adenocarcinoma (COAD) Data

COAD RNA-seq datasets (551 samples) from The Cancer Genome Atlas (TCGA) database was downloaded through the UCSC cancer genome browser (https://genome-cancer.ucsc.edu/, accessed on 17 March 2021). The box and whisker plot was constructed using GraphPad Prism. The corrplot function (R package corrplot) was used to confirm the correlation between the expression levels of IL-23A and other genes. Gene Microarray Analysis: All CRC gene microarray data was downloaded from the NCBI Gene Expression Omnibus (GSE103512; PMID 29133367). For IL23A expression, the probe (220054_PM_at) was quantified in healthy weight (<25 BMI) or overweight/obese patients (≥25 BMI), which were compared by the Mann–Whitney U test (*p* = 0.0362). For estimation of immune infiltrates in the sample, microarray probe IDs from GSE103512 were converted to their respective gene symbols and the probe with maximum expression among duplicate probes was retained for further analysis. The resulting dataset was used to perform analysis with TIMER 2.0 (PMID 32442275) to obtain estimates of immune infiltrates in each sample. The resulting infiltrate estimates were used for correlation analysis with IL23A gene expression.

### 2.4. Cell Lines

Human colon cancer cell lines (Caco2 (Lot number:70013347) and HCT116 (Lot number: 70019042) and monocyte THP-1 (Lot number: 70005912) cell lines were purchased from the American Type Culture Collection (ATCC, Rockville, MD, USA). Colon cancer cells were cultured in DMEM, supplemented with 10% FBS, 100 units/mL penicillin at 37 °C, and 5% CO_2_. Colon cancer cells were treated with 20, 40, and 100 ng/mL of recombinant human IL-23 (rhIL-23) for 24 h. After 24 h, cells were used for organoid culture, migration, invasion assays, and cell lysates were prepared for Western blotting analysis as detailed below. THP-1 cells were grown in RPMI complete medium as per the manufacturer’s recommendation. THP-1 cells were cultured and treated with 100 ng/mL of Phorbol-12-myristate-13-acetate (PMA) for 48 h to generate macrophages. To generate Dendritic cells (DCs), THP-1 cells were resuspended in culture medium supplemented with 10% FBS, rhGM-CSF (100 ng = 1500 IU/mL), rhIL-4 (100 ng = 1500 IU/mL) and cultured for 5 days. Every 2 days, a medium exchange was performed. THP-1 derived DCs and macrophages were educated with AA-50 μM, PGE_2_-10 μM, LTA-10 μg/mL and LPS-1 μg/mL for 24 h. The culture-conditioned medium was collected for ELISA analysis and cell lysates were prepared for Western blotting analysis. All cell lines were free of mycoplasma contamination, tested by PCR using specific oligonucleotides (IDT, Chicago, IL, USA), and authenticated by short tandem repeat profiling (ATCC).

### 2.5. Co-Culture of DCs/Macrophages with Cancer Cells

For co-culture experiments, DCs or macrophages were seeded in trans-well inserts (0.4 μm pore size) supplemented with or without AA, PGE_2_, LTA, and LPS. After stimulation, inserts were transferred to the plates containing Caco2/HCT116 cells for 24 h. The spent medium was collected for ELISA analysis and tumor cells were utilized for organoid culture, migration, invasion assays, and Western blotting analysis.

### 2.6. Cell Proliferation Assay

Cells were treated with 20, 40, and 100 ng/mL of rhIL-23 for 24 h. A total of 10 μL MTT solution was added to the 100 uL of the medium in each well and the cells were incubated at 37 °C for 2 h. Then, 200 μL of 266 mmol/L NH_4_OH in DMSO was added to the wells to dissolve the formazan salt. The absorbance was measured at 570 nm using a microplate reader and normalized by subtracting background absorbance at 630 nm. The results were expressed as absorbance at different doses of treatment.

### 2.7. Cell Migration and Invasion Assay

Matrigel-coated trans-wells were retrieved in serum-free media for 2 h at 37 °C and used for the invasion assay. For the migration assay, trans-wells were used. Colon tumor cells (1 × 10^4^) suspended in 2% FBS containing DMEM medium were seeded in the upper chambers (8 μm) and placed in a 24-well plate. The lower chamber was filled with 20% FBS containing DMEM media. Following incubation for 48 h at 37 °C under 5% CO_2_, the inserts were removed. A cotton swab was used to scrape non-invaded/migratory cells off the top of trans-wells. The remaining cells were fixed and stained with 0.1% crystal violet, rinsed with water, and allowed to dry. The invaded and migrated cells were determined by counting cells in four microscopic fields per well. The results were expressed as the average number of cells per field.

### 2.8. Organoid Culture

Tumor cells in RPMI medium containing 1% B27/N2, 1% FBS, EGF (50 ng/mL), Noggin (100 ng/mL) and R-spondin 1 (500 ng/mL) and Y-27632 (10 μM) were mixed with reduced growth-factor matrigel (1:1) [17]. A hundred microliters of the mixture were pipetted into 48-well ultra-low attachment plates at 5000 cells/well. The plates were then incubated at 37 °C under 5% CO_2_. Wells were monitored for organoid formation and were counted and photographed at 96 h as described previously [17,18]. The results were expressed as the number of organoids per well.

### 2.9. Enzyme-Linked Immunosorbent Assay (ELISA)

The quantitative determination of human IL-23 was carried out using an ELISA kit according to the manufacturer’s instructions. Results are expressed as pg/mL.

### 2.10. RNA Interference Studies

THP-1 monocytes-derived DCs and macrophages were transfected with 100 nM IL-23 siRNA and Scramble siRNA using viromer blue transfection reagent as per the manufacturer’s instructions. Thirty-six hours later, the cells were stimulated with or without AA, PGE_2_, LTA, and LPS and co-cultured with tumor cells. After 24 h, the conditioned medium and tumor cells were collected for further analyses.

### 2.11. Ex Vivo Assay

Seven weeks old male F344 rats were fed the modified AIN-76A diet for a week, after which colon carcinogen AOM was administered by subcutaneous injections at a dose of 15 mg/kg body weight once weekly for two weeks. The experiment was terminated 48 weeks after the second AOM treatment, at which time all animals were euthanized via CO2 euthanasia. Colon tumor tissues and mucosa from AOM induced rats were equally placed in various wells and exposed to AA and PGE_2_ in DMEM supplemented with 10% FBS for 1 h at 37 °C under 5% CO_2_. After 1 h, tumor tissues and mucosa were harvested and processed for Western blotting analysis.

### 2.12. Real-Time-PCR Analysis

Total RNA was isolated from the immune cells using Trizol and was subjected to reverse transcription using an iScript cDNA synthesis kit and the complementary DNA (cDNA) was subsequently used to perform real-time (RT)-PCR (Bio-Rad CFX96 Touch Real-Time PCR Detection System) with SYBR chemistry using iQTM SYBR Green supermix and using human IL-23A-specific oligonucleotide primers. The crossing threshold (Ct) value assessed by RT-PCR was noted for the transcripts and normalized with human 18S mRNA. The changes in mRNA were expressed as fold change relative to control ± the standard deviation (SD).

### 2.13. Immunoblot Analysis

Cell and tissue lysates were prepared and total protein concentration was determined by BCA protein assay. Protein extracts (30–60 μg protein/lane) were subjected to SDS polyacrylamide gel and electro-transferred onto a PVDF membrane with a wet-blot transfer apparatus (Bio-Rad, Hercules, CA, USA). The membranes were blocked and incubated overnight with primary antibodies and were subsequently incubated with horseradish peroxidase-conjugated appropriate secondary antibodies. The protein expressions were detected using ECL Western blotting detection reagents. Beta-actin was used as an internal loading control. Protein density quantification was performed using GelQuant software.

### 2.14. Immunofluorescence

THP-1 derived DCs (1 × 10^4^) were allowed to adhere to poly-L-lysine-coated coverslips for 5 min by cytospin and fixed in 4% paraformaldehyde in PBS for 10 min at room temperature (RT). After fixation, the cells were washed with PBS followed by incubation in blocking buffer (5% BSA in PBS) for 30 min at RT. Then, a DC-SIGN antibody was added in blocking buffer and incubated at 4 °C overnight. The cells were subsequently washed with PBS, followed by the addition of Alexa Fluor^®^ 488 conjugated secondary antibody diluted in the blocking buffer. The cells were incubated in the dark for 1 h at RT, then counterstained with DAPI for 5 min. The stained cells were washed with 1× PBS, mounted with ProLong Gold, and examined. Photographs were captured using a Nikon TiU microscope (Nikon Instruments Inc., Melville, NY, USA). For tissue slides: Slides were incubated in normal serum and BSA blocking step at room temperature for 20 min. After incubation with primary antibody overnight at 4 °C, slides were labeled with Alexa Fluor dye–conjugated secondary antibody and mounted with ProLong Gold (Invitrogen). Image Acquisition: Slides were examined and photographs were captured using a Nikon TiU microscope (Nikon Instruments Inc., Melville, NY, USA).

### 2.15. Statistical Analysis

All statistical analyses were performed using GraphPad Prism 8.4.3 and Microsoft Excel. One-way ANOVA followed by Tukey’s and Newman–Keuls Test were performed and the Student’s *t*-test was used to determine statistical significance. Pearson product-moment correlation was used for analysis and correlation of gene expressions between two groups. Colon cancer disease-free survival analysis was performed using Kaplan Meier Survival analysis. All assays were replicated a minimum of three times. *p* values of <0.05 = *, <0.01 = **, <0.001 = *** were considered statistically significant.

## 3. Results

### 3.1. IL-23 Expression Correlates with Disease Stage, Disease-Free Survival, and Obesity in Colon Cancer

**Figure 1:**
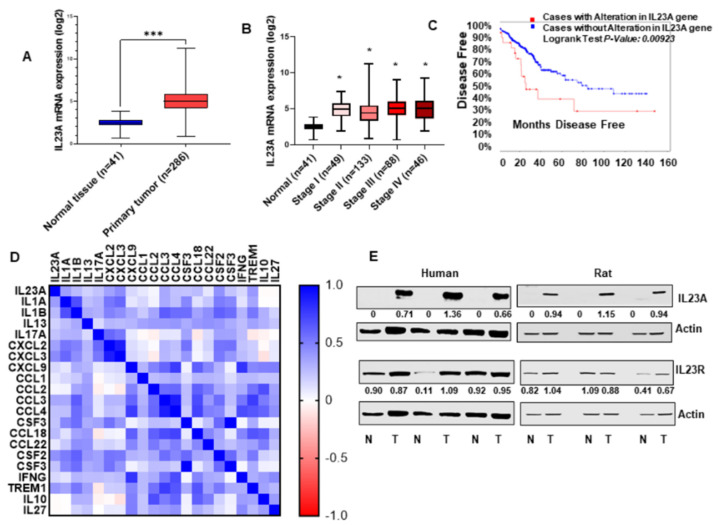
IL-23A expression in human colon adenocarcinoma. (**A**) The TCGA COAD database of 551 patients showed a higher mRNA expression of IL-23A in the primary tumor samples than in normal solid tissues. (**B**) The pathological stage of the COAD dataset demonstrated that mRNA expression of IL-23A is highly upregulated in all stages of colorectal adenocarcinoma, compared with normal solid tissues. (**C**) Kaplan–Meier survival curve analysis showed that patients with high IL-23A gene expression had low disease-free survival compared with patients with low IL-23A gene expression. (**D**) IL-23A mRNA and mRNA of cytokines and chemokines were downloaded from the COAD dataset of the TCGA database. The difference in the color indicates a correlation of IL-23A with other genes, positive (blue) and negative (red). (**E**) IL-23A and IL-23R protein expressions in human and rat, colon tumors and their matched normal colon tissues. Band intensity ratio was measured using GelQuant software. * *p* < 0.05, and *** *p* < 0.001 were considered statistically significant.

To determine IL-23A expression in colon cancer patient’s tumors, we analyzed the IL-23A gene expression data from the TCGA COAD database. We observed that IL-23A mRNA expression is higher in the primary tumor samples than in the normal tissues (*p* < 5.63995E-26) (Figure 1A). Furthermore, IL-23A expressions were highly increased across all the stages of colon cancer as compared to normal tissues (Figure 1B). However, IL-23A expression between the four stages (I, II, III, IV) of colon cancer is not significantly altered (Figure 1B). *Kaplan–Meier* survival curve analysis showed that cases with increased expression of IL-23A had lower disease-free survival rates compared to cases with low IL-23A expression (*p* < 0.0501) (Figure 1C). TCGA-COAD database analysis also revealed an association between IL-23A expression and body weight in colon cancer patients (normal vs obese; *p* < 2.656100E-02) (Figure S1A). TCGA-COAD database was utilized for the correlation analysis between IL-23A and pro-inflammatory cytokines/chemokines. Our analysis revealed that IL-23A is strongly correlated with the expression of pro-inflammatory cytokines, IL-1A, IL-1B, IL-13, IL-17A, CXCL-2, CXCL-3, CXCL-9, CCL-1, CCL-3, CCL-4, CCL-18, CSF-2, CSF-3, IFNG, TREM-1, and weak correlation with anti-inflammatory cytokines such as IL-10 and IL-27 expression in colon cancer (Figure 1D). IL-23 is significantly upregulated in obese/overweight patients compared to healthy weight patients, and also IL-23 is positively correlated with myeloid dendritic cells (Figure S1B). Furthermore, we stained IL-23 in the rat colonic tumor tissues co-stained with DC-sign. We found that IL-23 is co-expressed with DC-sign marker suggests that IL-23 is secreted by dendritic cells (Figure S1C). We observed a significant increase in the expression level of IL-23A and its receptor IL-23R in colon cancer tissues of humans, and AOM treated rat colon cancer model when compared with matched normal mucosa (Figure 1E; Figure S11). Together, our data indicate that IL-23 increased in colon cancer and strongly correlated with pro-inflammatory cytokines/chemokines, obesity, disease stage, and poor disease-free survival.

### 3.2. IL-23 Promotes Colon Tumor Cell Proliferation

**Figure 2:**
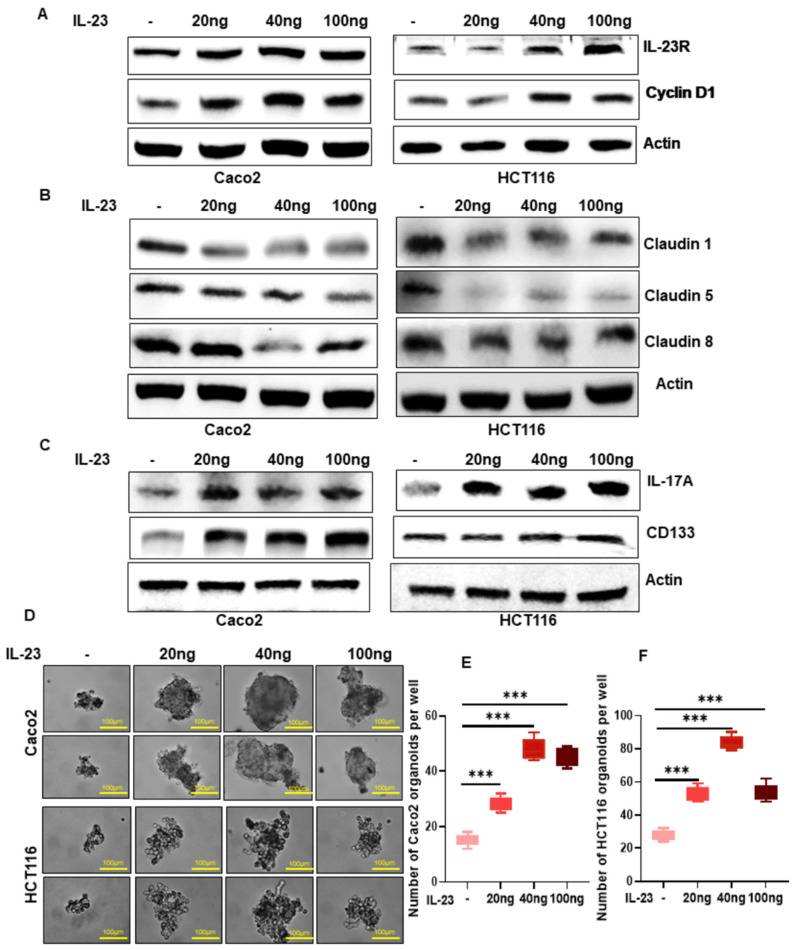
Effect of IL-23 on colon tumor cell proliferation, epithelial barrier integrity, and stemness. (**A**) Western blotting analysis showed that treatment of rhIL-23 in colon tumor cells increased the expression of IL-23R and cyclin D1. (**B**) Western blotting analysis showed the effect of rhIL-23 treatment on the expression of claudin1, 5, and 8 in colon tumor cells. (**C**) Expression of IL-17A and CD133 in colon tumor cells upon treatment with rhIL-23. Beta-actin was used as a protein loading control. (**D**) Treatment of rhIL-23 increased the number of organoids compared with untreated control cells (Magnification 40×). (**E**,**F**) Quantification of organoids in control and rhIL-23 treated cells. All experiments were performed a minimum of three times. Bars denote standard deviation (SD). *** *p* < 0.001 were considered statistically significant.

To study the direct effect of IL-23 on colon cancer cells, we treated Caco2 and HCT116 cell lines with different concentrations (20, 40, and 100 ng) of rhIL-23. We found that the expression of IL-23R was increased in Caco2 in response to rhIL-23 treatment at all tested doses (Figure 2A; Figures S2A and S11). However, in HCT116 cells, rhIL-23 at 40 ng and 100 ng increased the expression of IL-23R (Figure 2A; Figure S2A). Treatment of colon cancer cell lines with rhIL-23 increased the expression of the cell proliferation marker cyclin D1 in Caco2 cells at all doses, however, in HCT116 only 40 and 100 ng doses increased the expression of cyclin D1 (Figure 2A; Figure S2A). We observed increased proliferation of Caco2 and HCT116 cells after rhIL-23 treatment (Figure S3A). Although Caco2 and HCT116 cell proliferation was increased at all concentrations of rhIL-23 treatment, these cell lines displayed a better response at 40 and 100 ng.

### 3.3. IL-23 Reduced the Integrity of Tumor Epithelial Tight Junction

The epithelial barrier integrity loss potentially contributes to colon tumorigenesis. Claudins are tight junctional proteins and their dysregulation has been shown to modulate barrier permeability, inflammation, and tumorigenesis in the gastrointestinal tract [19]. To evaluate the effect of IL-23 in colon tumor epithelial cell permeability we analyzed the expression of claudins 1, 5, and 8. Treatment of rhIL-23 reduced the expression of claudins 1, 5, and 8 particularly at 40 and 100 ng concentration in Caco2 cells compared to vehicle-treated controls (Figure 2B; Figures S2B and S11). Treatment of rhIL-23 at 20 ng showed no marked change in claudin 8 expression in Caco2 cells (Figure 2B; Figure S2B). Likewise, IL-23 treatment significantly decreased the expression of claudin 1, 5, and 8 protein in HCT116 cells compared to vehicle-treated cells (Figure 2B; Figure S2B). Our data suggest that IL-23 can directly impair the epithelial barrier permeability in the colon tumor and maybe in the epithelium for tumor growth and progression. (Figure 2B).

### 3.4. IL-23 Increases Organoid Formation, Migration, and Invasion of Colon Cancer Cells

Stemness, self-renewal (organoid formation), migratory, and invasive abilities are the key features in tumorigenesis, for tumor initiation and progression [20]. Earlier studies reported that IL-23 via its effector molecule IL-17A induces the self-renewal ability of tumor cells [21]. We observed an increase in the expression of IL-17A in both Caco2 and HCT116 cells after the treatment of rhIL-23 at all concentrations (Figure 2C; Figures S2C and S11). CD133, a cancer stem cell marker and confers malignant stemness [22], is upregulated in Caco2 and HCT116 cells with 40 and 100 ng rhIL-23 treatment compared to vehicle-treated cells (Figure 2C; Figure S2C). However, the expression of CD133 in HCT116 cells was not increased at 20 ng rhIL-23 treatment compared to vehicle-treated cells.

To further understand the role of IL-23 on colon tumor cell self-renewal ability, we cultured tumor cells with and without rhIL-23 for 24 h, and cells were collected for a matrigel 3D culture system. The organoid formation in the 3D culture was monitored every 24 h and the number of organoids were counted at 96 h. We observed that IL-23 increased the number of organoids at all doses compared to control groups (Figure 2D–F). Indeed, the number of organoids was higher at 40 ng of rhIL-23 treatment. Our finding demonstrates that IL-23 promotes the self-renewal ability of colon tumor cells, which is an important characteristic of cancer stem cells for tumor progression [20,23]. Interestingly, the treatment of rhIL-23 (effective dose 40 ng) significantly increased the migratory and invasive ability of Caco2 and HCT116 cells compared with the vehicle-treated control group (Figure S3B). Taken together, this data indicates that IL-23 can promote colon cancer progression through enhancing cell self-renewal/stemness, migratory, and invasive ability.

### 3.5. Effect of AA, PGE_2,_ and Bacterial Toxins on IL-23 Production in Dendriticcells

**Figure 3:**
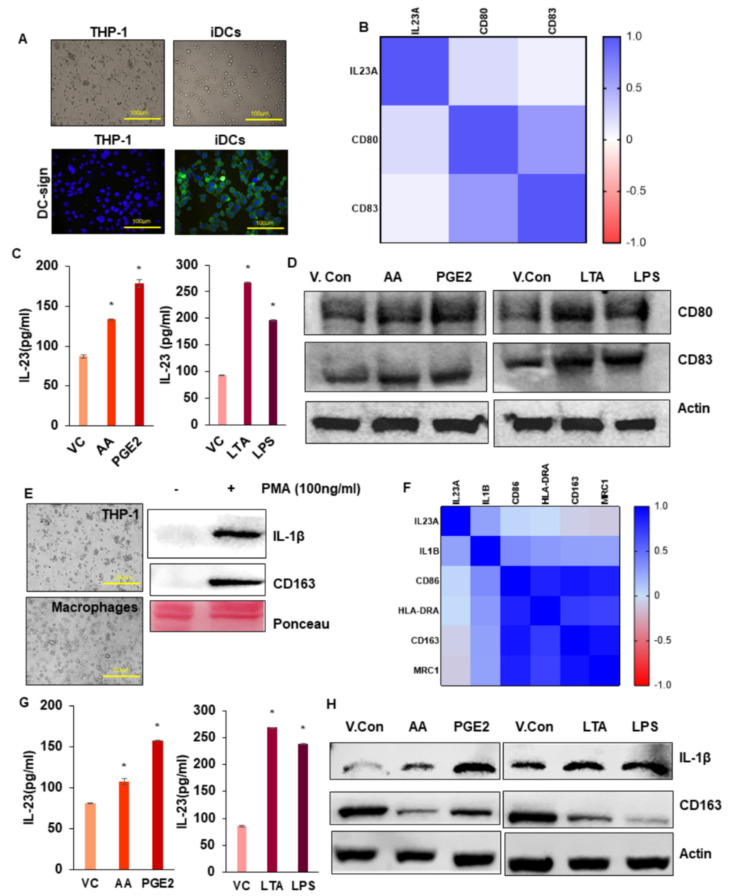
Effect of obesity-associated factors and bacterial toxins on IL-23 production in dendritic cells and macrophages. (**A**) Morphology (Magnification 10×) and immunofluorescence staining (Magnification 60×) of DC-sign expression in THP-1 monocytes, and immature dendritic cells. (**B**) Analysis of correlation between IL-23A with pro-tumorigenic DC marker gene expressions using the TCGA-COAD RNA-seq database. (**C**) Quantification of IL-23 level in DCs upon treatment with or without AA, PGE_2_, LTA, and LPS by ELISA. (**D**) Western blotting analysis of CD80, CD83 expression in THP-1 monocyte-derived DCs upon treatment with or without AA, PGE_2_, LTA, and LPS. (**E**) Morphology of THP-1 monocytes, macrophages (Magnification 10X), and Western blotting analysis of Macrophage markers. (**F)** Analysis of correlation between IL-23A with macrophage markers gene expressions using TCGA-COAD RNA-seq database. (**G**) Quantification of IL-23 level in macrophages upon treatment with or without AA, PGE_2_, LTA, and LPS by ELISA. (**H**) Western blotting analysis of IL-1β and CD163 expression in THP-1 monocyte-derived macrophages upon treatment with or without AA, PGE_2_, LTA, and LPS. Beta-actin was used as a protein loading control. All experiments were performed a minimum of three times. Bars denote standard deviation (SD). * *p* < 0.05 was considered statistically significant.

DCs generated from THP-1 monocytes were confirmed by both morphology and the expression of DC-sign marker by immunofluorescence staining (Figure 3A). DCs represent a special group of immune cells that display two different phenotypes as pro-tumorigenic and anti-tumorigenic based on their phenotype maturation ligands (CD80-high, CD83-high) and the expression of IL-23 [24,25]. The expression of IL-23 (IL-23+) in a DC, along with the higher expression of phenotype maturation ligands, represents pro-tumorigenic phenotype which is involved in cancer progression and immune-suppression as compared to IL-23 negative (IL-23-) phenotype [24]. We analyzed the potential correlation between IL-23A with pro-tumorigenic DC marker gene expressions using the TCGA-COAD RNA-seq database. The dataset revealed that elevated IL-23A expression was positively correlated with CD80 and CD83 (Figure 3B). In this study, we investigated whether obesity-associated pro-inflammatory molecules and microbial toxins can polarize DCs into a pro-tumorigenic phenotype. We observed that the treatment of AA, PGE_2_, LTA, and LPS induces myeloid-derived DCs into a pro-tumorigenic DC phenotype with the expression of CD80-high, CD83-high, and increased IL-23 levels compared to vehicle-treated DCs with the expression of CD80-low, CD83-low, and low IL-23 level (Figure 3C,D; Figures S4A and S11).

### 3.6. Effect of AA, PGE_2,_ and Bacterial Toxins on IL-23 Production in Macrophages

Macrophages generated from THP-1 monocytes and were confirmed by morphological appearance as well as by the expression of macrophage markers (IL-1β, CD163) (Figure 3E; Figure S11). Macrophages based on their microenvironment can be converted into tumor-associated macrophages (TAMs), which have served as a paradigm for the connection between inflammation and cancer [26]. TAM influences all aspects of tumor growth and progression [27]. Cytokines play a key role in the tumor-promoting functions of TAM, in particular interest, expression of IL-23, and IL-1 induces TAMs for tumor progression [28]. We analyzed the correlation of IL-23A with macrophage marker gene expressions using the TCGA-COAD RNA-seq data. The dataset showed a strong correlation of increased expression of IL-23A with increased macrophage markers (IL-1β and HLA-DRA), which represents TAMs of M1 origin and with decreased macrophage markers (CD163, MRC1), which represent TAMs of M2 origin (Figure 3F) [26,28]. In this study, we investigated whether obesity-associated pro-inflammatory molecules and microbial toxins can polarize macrophages into a pro-tumorigenic TAM phenotype. We observed that the treatment of AA, PGE_2_, LTA, and LPS induces myeloid-derived macrophages into a pro-tumorigenic TAM phenotype with the increased IL-23 and IL-1β levels compared to vehicle-treated macrophages (Figure 3G,H; Figure S4B). Furthermore, Western blot analysis also confirmed that treatment of macrophages with AA, PGE_2_, LTA, and LPS decreased the expression of CD163 (Figure 3H; Figure S4B), suggesting that inflammatory factors including bacterial toxins induce TAMs of M1 origin. (Figure 3H; Figure S11).

### 3.7. IL-23 Production by Pro-Tumorigenic DCs/Macrophages Enhances Colon Tumor Cell Aggressiveness

**Figure 4:**
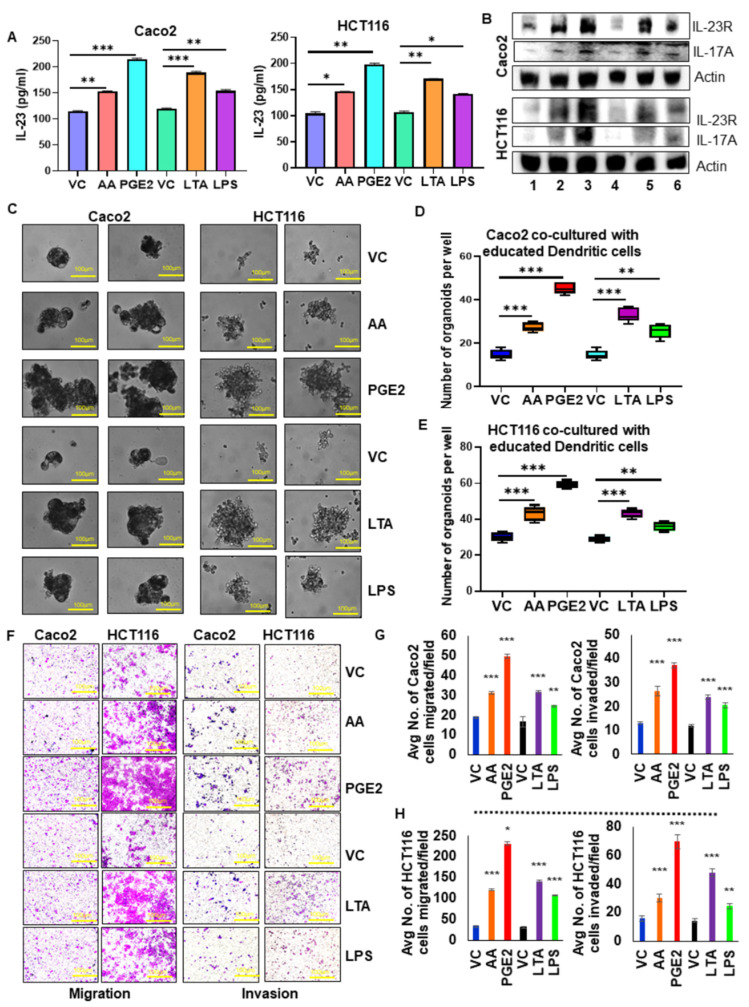
IL-23 production by pro-tumorigenic dendritic cells enhances colon tumor cell aggressiveness. (**A**) The level of IL-23 in the spent media of the co-culture system (Caco2/HCT116 + educated DCs with AA/PGE_2_/LTA/LPS) was measured using ELISA. (**B**) The expression of IL-23R, IL-17A were analyzed in Caco2 and HCT116 cells co-cultured with educated DCs as compared to uneducated DCs. Lane1-Vehicle control, Lane2-AA, Lane3- PGE_2_, Lane4- Vehicle control, Lane5- LTA, Lane6- LPS. Beta-actin was used as a protein loading control. (**C**) Co-culture of educated DCs with tumor cells increased the self-renewal ability of cancer cells compared with uneducated DCs co-culture system (Magnification 40×). (**D**,**E**) Quantification of organoids formed by tumor cells co-cultured with educated DCs compared to uneducated DCs. (**F**) Migration and invasion assay showed that tumor cells co-cultured with educated DCs increased migration and invasion compared to uneducated DCs (Magnification 10×). (**G**,**H**) Quantification of the number of migrated and invaded cells. All experiments were performed a minimum of three times. Bars denote standard deviation (SD). * *p* < 0.05, ** *p* < 0.01, and *** *p* < 0.001 were considered statistically significant.

To elucidate the pro-tumorigenic role of educated DCs in the tumor microenvironment, we co-cultured educated DCs with tumor cells in an in vitro model system using trans-wells. DCs were educated with PGE_2_ or AA or LTA or LPS and used for the co-culture system. Caco2 or HCT116 colon tumor cells were co-cultured with educated DCs and evaluated for IL-23 levels in the spent media. We found a significant increase in the level of secretory IL-23 protein in the spent media of the co-culture system with educated DCs compared to uneducated DCs. (Figure 4A). Moreover, we observed a marked increase in the expression of IL-23R and IL-17A in both cancer cell lines when co-cultured with educated DCs as compared to uneducated DCs (Figure 4B; Figures S5A,B and S11). We observed a marked increase in the self-renewal ability of cancer cells when co-cultured with educated DCs (Figure 4C–E). Further, we also observed a significant increase in tumor cell migration and invasion when co-cultured with educated DCs compared to uneducated DCs (Figure 4F–H).

**Figure 5:**
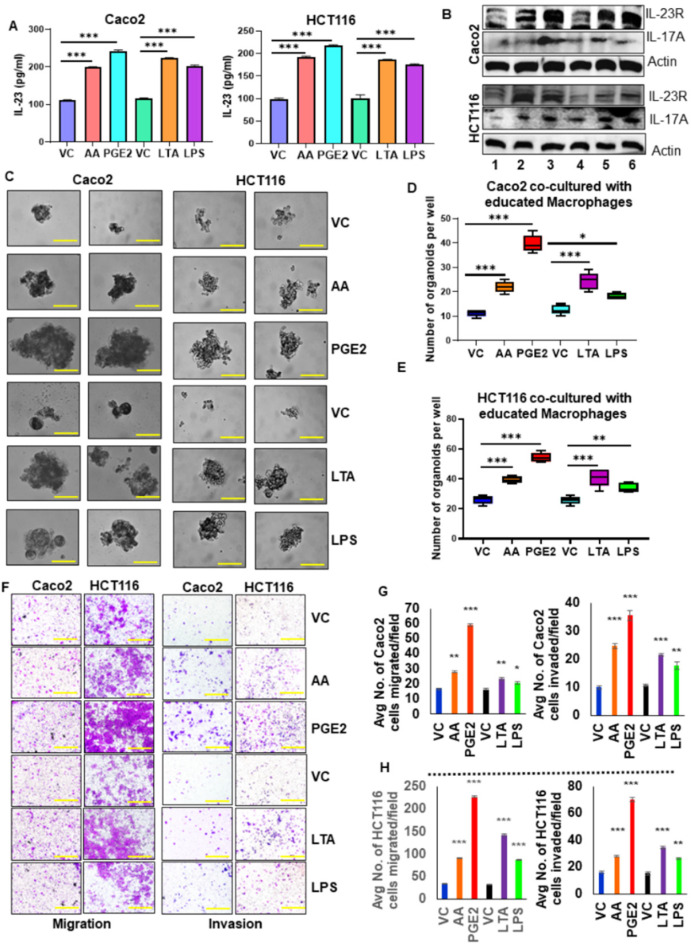
IL-23 production by macrophages enhances colon tumor cell aggressiveness. (**A**) The level of IL-23 in the spent media of the co-culture system (Caco2/HCT116 + educated macrophages with AA/PGE_2_/LTA/LPS) was measured using ELISA. (**B**) The expression of IL-23R, IL-17A were analyzed in Caco2 and HCT116 cells co-cultured with educated macrophages compared to uneducated macrophages. Lane1-Vehicle control, Lane2-AA, Lane3- PGE_2_, Lane4- Vehicle control, Lane5- LTA, Lane6- LPS. β-actin was used as a protein loading control. (**C**) Co-culture of educated macrophages with tumor cells increased the self-renewal ability of cancer cells compared with uneducated macrophages co-culture system (Magnification 40×). (**D**,**E**) Quantification of organoids formed by tumor cells co-cultured with educated macrophages compared to uneducated macrophages. (**F**) Migration and invasion assay showed that tumor cells co-cultured with educated macrophages increased migration and invasion compared to uneducated macrophages (Magnification 10×). (**G**,**H**) Quantification of the number of migrated and invaded cells. All experiments were performed a minimum of three times. Bars denote standard deviation (SD). * *p* < 0.05, ** *p* < 0.01, and *** *p* < 0.001 were considered statistically significant.

To elucidate the tumorigenic role of educated macrophages in the tumor microenvironment, we co-cultured educated macrophages with tumor cells in an in vitro model system using trans-wells. Macrophages were educated with PGE_2_ or AA or LTA or LPS and used for the co-culture system. Caco2 or HCT116 colon tumor cells were co-cultured with educated macrophages and evaluated for IL-23 levels in the spent media. We found a significant increase in the level of secretory IL-23 protein in the spent media of the co-culture system with educated macrophages compared to uneducated macrophages (Figure 5A). Moreover, we observed a marked increase in the expression of IL-23R and IL-17A in both cancer cell lines when co-cultured with educated macrophages as compared to uneducated macrophages (Figure 5B; Figures S6A,B and S11). The self-renewal ability of cancer cells was increased when co-cultured with educated macrophages (Figure 5C–E). Further, we also noted a significant increase in tumor cell migration and invasion when co-cultured with educated macrophages compared to uneducated macrophages (Figure 5F–H).

Ex vivo models represent an efficient and accurate preclinical tool to investigate the interaction of tumor cells and inflammatory cells in their microenvironment as it includes all cell types including, tumor epithelial cells, stromal and immune cells. To confirm our data obtained above, an ex vivo analysis was performed using AOM-induced rat colon tumors and adjacent normal mucosa. Treatment of AA and PGE_2_ to ex vivo colon tumors significantly increased the expression of IL-23 compared to adjacent normal mucosa (Figure S7). However, between AA and PGE_2_, IL-23 expression is increased more by PGE_2_ than AA in ex vivo colon tumors. These results confirmed our in vitro data that the obesity-associated pro-inflammatory molecules can induce or influence the IL-23 production within the colonic tumor environment for tumor progression.

### 3.8. Inhibition of IL-23 in Immune Cells Reduces Colon Cancer Self-Renewal, Migratory and Invasion Ability

**Figure 6:**
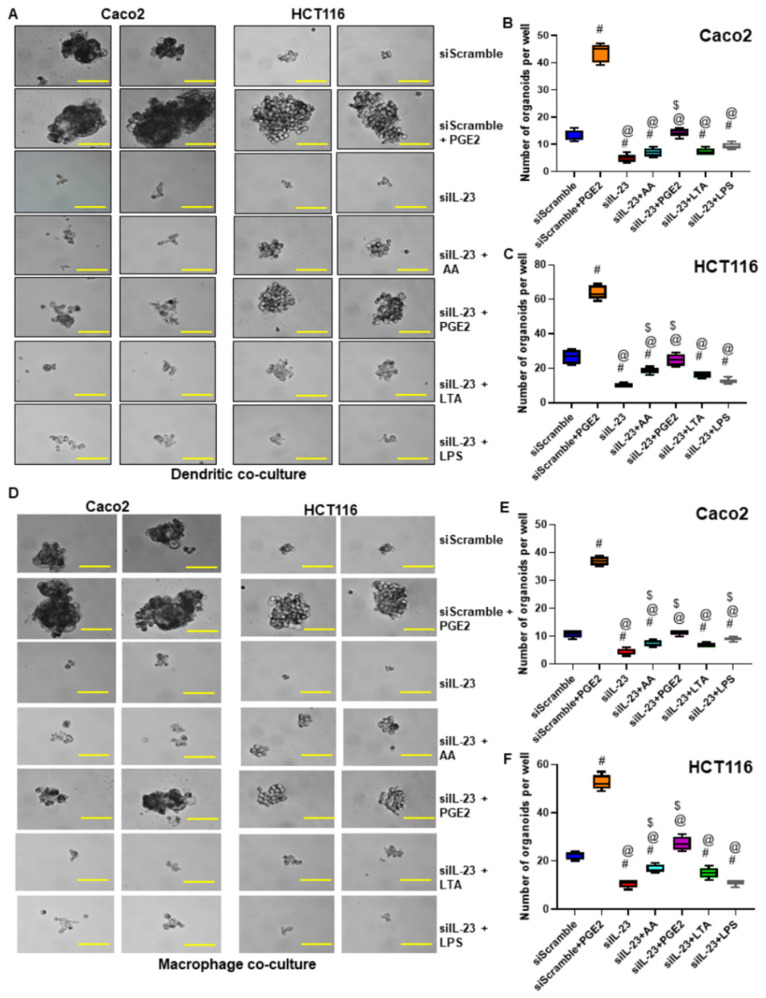
Inhibition of IL-23 in DCs and macrophages reduced colon tumor cell self-renewal. (**A**,**D**) Effect of siRNA knockdown of IL-23 in educated DCs and macrophages on the self-renewal ability of co-cultured Caco2 and HCT116 cells compared to scramble siRNA and scramble siRNA + PGE_2_ stimulated immune cells (Magnification 40×). (**B**,**C**,**E**,**F**) Quantification of organoids formed per well by tumor cells co-cultured with siIL-23 treated and educated DCs and macrophages compared to scramble siRNA treated and uneducated macrophages. #-compared with siScramble; @-compared with siScramble + PGE_2_; $-compared with siIL-23. All experiments were performed a minimum of three times. Bars denote standard deviation (SD).

Next, we investigated whether educated immune cells secreting IL-23 is critical for tumor aggression. We knocked down IL-23 in the THP-1 derived DCs and macrophages using small interference RNA against IL-23 (siIL-23). Knockdown of IL-23 in the immune cells was confirmed by RT-PCR analysis (Figure S8). The siIL-23 treated DCs and macrophages were educated with or without AA, PGE_2_, LTA, and LPS and used to co-culture with tumor cells. After 24 h of co-culture, the tumor cells were utilized for self-renewal assay. We observed that silencing IL-23 in the DCs or macrophages completely limits their ability to induce self-renewal of the tumor cells compared to siScramble treatment. (Figure 6A–F). Interestingly, silencing IL-23 in the AA, LTA and LPS educated DCs or macrophages inhibits their ability to induce tumor cell self-renewal compared to siScramble treated and positive control (PGE_2_) treated immune cells (Figure 6A–F). However, PGE_2_ educated DCs or macrophages with silenced IL-23 co-cultured with tumor cells showed a moderate ability to induce tumor cell self-renewal compared to siScramble and siIL-23 treatment. (Figure 6A,D). IL-23 knockdown in the DCs and macrophages inhibited their secretory ability of IL-23 even after being stimulated with AA, LTA, and LPS (Figure S9). Furthermore, we demonstrated that inhibition of IL-23 in educated immune cells decreased the migratory and invasive ability of tumor cells compared to siScramble and si-Scramble + PGE_2_ stimulated immune cells (Figure S10A–D). PGE_2_ has moderately induced the secretory IL-23 in the IL-23 inhibited DCs and macrophages. However, further molecular analysis using a complete knockout of IL-23 is required to identify the mechanism of PGE_2_ regulated IL-23 in the immune cells associated with obesity. Taken together, these studies have demonstrated the important function of immune cells in an obese environment to promote and progress colon cancer in an IL-23 dependent mechanism.

## 4. Discussion

WSD intake is more common in America and is now increasing worldwide. It is suspected to be a cause for the obesity endemic by modulating various inflammatory pathways. Accumulating epidemiological and preclinical studies show that WSD-induced obesity is one of the leading risk factors in the development of colon cancer [29]. An increasing number of studies implicate that chronic inflammation plays a key role in WSD induced obesity-associated colon cancer [30]. It is reported that the intake of WSD alters the gut microbiome with a reduced ratio of *Bacteroides* to *Firmicutes* [31]. WSD induced dysbiosis of the gut microbiome is suspected to increase gut permeability by decreasing the expression of tight junctional proteins and is associated with colonic inflammation [32]. WSD-induced dysbiosis is proposed to be another important factor influencing the colonic microenvironment toward an inflammatory environment to facilitate colon cancer [33]. However, there is a need to understand the fundamental gap in the interface of molecular mechanisms between WSD-induced obesity, gut bacteria, and inflammation in colon cancer development and progression. Recent studies have demonstrated that innate immune cells, predominantly macrophages and dendritic cells, show tumor-promoting effects on neoplastic progression [34]. IL-23 is primarily produced by dendritic cells and macrophages and is suggested to be a key factor in chronic inflammation-mediated colon cancer [35]. It is reported that treatment of IL-23 increased esophageal and thyroid cancer proliferation and EMT [36,37]. IL-23 may be a common link in obesity-associated altered gut microbiota, and chronic inflammation-induced colon cancer development and progression. However, the precise mechanistic link between obesity-mediated change in immune cell phenotypes and the associated increase in the IL-23 for colon tumorigenesis and progression is not clear. Our study showed that IL-23 is highly expressed in human and rodent colon tumor samples. Its expression is also strongly correlated with BMI suggesting that a circulating level of IL-23 is highly increased under obese conditions. In accordance, it is reported that IL-23A is increased in colon cancer [38]. It is also reported that IL-23 triggers an inflammatory cascade through the expansion of the Th17 population [39]. TCGA database analyses revealed that IL-23A expression in human colon cancer is strongly correlated with pro-inflammatory molecules and weakly correlated with anti-inflammatory factors. Previous study results showed that IL-23 is increased in inflammatory bowel disease and it contributes to the activation of immune cells to promote CRC [40]. Our data is consistent with these studies and strongly suggests that increased IL-23 is a pro-inflammatory milieu that contributes to the progression of colon cancer.

High-grade colon cancers are poorly differentiated tumors with aggressive tumor features including invasive ability and stemness properties [41]. Our findings suggest that IL-23 can directly enhance the aggressiveness of colon cancer, facilitating cancer cells into a high-grade phenotype by increasing cancer cell proliferation, migration, invasion, and self-renewal. In accordance with the present study, a recent report strongly suggests that IL-23, directly or via IL-17, enhances tumor stemness [42]. Loss of epithelial barrier function can cause unbalanced immune activation and chronic inflammation in the colon. Claudin family proteins are considered essential for the integrity of the intestinal barrier and dysregulated claudins were involved in the loss of epithelial barrier function and aberrant activation of immunity and inflammation results in colon cancer development and progression [43]. It is reported that IL-23 downregulates CLDN8 in both IBD patients and mice with colitis by upregulating miR223 [44]. Our findings demonstrated that IL-23 directly dysregulates the epithelial integrity by downregulating claudin proteins in the colonic cancer cells suggesting its role in cancer progression.

Obesity-induced inflammation is considered primarily an innate immune response. Obesity is a significant driver for the composition of gut microbiota in promoting obesity-associated colon cancer [45]. Increasing evidence suggests that the altered gut microbiota composition together with the host immune system-mediated pro-inflammation are primarily involved in colon tumor development [46]. DCs and macrophages represent the majority of innate immune cells whose population is known to increase by nearly 10-fold in obese conditions [47]. These immune cells are the predominant sources of the pro-inflammatory cytokine IL-23 [48,49,50]. Our study explored the mechanistic importance of pro-inflammatory mediators (AA and PGE_2_) and bacterial toxins (LTA and LPS) in priming the DCs and macrophages into a pro-tumorigenic phenotype and the production of IL-23. In support of our in vitro study, colon tumor ex vivo studies, which represent tumors with the tumor microenvironment, confirmed that treatment of AA and PGE_2_ increased the IL-23 production. Furthermore, we demonstrated that the generated/educated pro-tumorigenic DCs and macrophages facilitate colon cancer high-grade progression by enhancing colon cancer cell migration, invasive, and self-renewal ability. Interestingly, when IL-23 was knocked down in the DCs and macrophages, their interaction with the tumor cells even after being educated with pro-inflammatory mediators (AA and PGE_2_) and bacterial toxins (LTA and LPS) did not support tumor aggression. Taken together our results demonstrate that obesity-mediated pro-tumorigenic DCs and macrophages facilitate colon cancer progression in an IL-23 dependent mechanism.

## 5. Conclusions

Our study results demonstrate that obesity-associated inflammatory mediators (AA and PGE_2_) and gut microbe toxins (LTA and LPS) polarize DCs and macrophages into a pro-tumorigenic phenotype to facilitate colon cancer progression. Our findings signify the importance of IL-23 production by the pro-tumorigenic DCs and macrophages in the process of tumorigenesis. This newly identified nexus provides a strong rationale to target IL-23 for the prevention and treatment of obesity-associated colon cancer initiation and progression.

## Data Availability

Data is contained within the article or Supplementary Material.

## Supplementary Materials

The following are available online at https://www.mdpi.com/article/10.3390/cancers13205159/s1, Figure S1: A: The colon cancer samples downloaded from the TCGA database demonstrated the positive association between the expression of IL-23A and patient body weight; B: All CRC gene microarray data was downloaded from the NCBI Gene Expression Omnibus. IL-23 expression was compared with healthy and overweight/obese patients and demonstrated the positive association between the expression of IL-23A and myeloid cell markers; C: IHC: staining of DC-sign co-stained with IL-23 in the rat colon cancer tissues; Figure S2: A,B and C: Bar graph represent the semi-quantitative analysis of protein expression. Error bar denotes SD. * *p* < 0.05, ** *p* < 0.01, and *** *p* < 0.001; Figure S3: A: Effect of rhIL-23 on cell proliferation of colon tumor cells. * *p* < 0.05, ** *p* < 0.01, and *** *p* < 0.001; B: Effect of rhIL-23 on migration and invasion of colon tumor cells. * *p* < 0.05, ** *p* < 0.01, and *** *p* < 0.001; Figure S4: A and B: Bar graph represent the semi-quantitative analysis of protein expression. Error bar denotes SD. * *p* < 0.05, ** *p* < 0.01, and *** *p* < 0.001; Figure S5: A and B: Bar graph represent the semi-quantitative analysis of protein expression. Error bar denotes SD. * *p* < 0.05, ** *p* < 0.01, and *** *p* < 0.001; Figure S6: A and B: Bar graph represent the semi-quantitative analysis of protein expression. Error bar denotes SD. * *p* < 0.05, ** *p* < 0.01, and *** *p* < 0.001; Figure S7: Ex vivo analysis of the effect of AA and PGE2 on IL-23 expression in rat mucosa and colonic tumor; Figure S8: THP-1 derived macrophages and dendritic cells were transfected with siIL-23 and knockdown of IL-23 expression was confirmed by RT-PCR analysis; Figure S9: Effect of siRNA knockdown of IL-23 in educated DCs and macrophages on the level of IL-23 in the spent media of co-culture system (Caco2/HCT116+educated DCs/macrophages with AA/PGE2/LTA/LPS) was measured using ELISA; Figure S10: Inhibition of IL-23 in DCs and macrophages reduced colon tumor cell migration and invasion. (A,B) Effect of siRNA knockdown of IL-23 in educated DCs and macrophages on the cell migration and invasion ability of co-cultured Caco2 and HCT116 cells compared to scramble siRNA and scramble siRNA+PGE2 stimulated immune cells. (C,D) Quantification of the number of migrated and invaded tumor cells per field co-cultured with siIL-23 treated and educated DCs and macrophages compared to scramble siRNA treated and uneducated macrophages. #—compared with siScramble; @—compared with siScramble+PGE2; $—compared with siIL-23; Figure S11: Original blots; Table S1: Reagent Sources.
