# Correction: Panneerselvam et al. Inflammatory Mediators and Gut Microbial Toxins Drive Colon Tumorigenesis by IL-23 Dependent Mechanism. *Cancers* 2021, *13*, 5159

**DOI:** 10.3390/cancers17030352

**Published:** 2025-01-22

**Authors:** Janani Panneerselvam, Venkateshwar Madka, Rajani Rai, Katherine T. Morris, Courtney W. Houchen, Parthasarathy Chandrakesan, Chinthalapally V. Rao

**Affiliations:** 1 Center for Cancer Prevention and Drug Development, The University of Oklahoma Health Sciences Center, Oklahoma City, OK 73104, USAvenkateshwar-madka@ouhsc.edu (V.M.); 2 Stephenson Cancer Center, The University of Oklahoma Health Sciences Center, Oklahoma City, OK 73104, USA; rajani-rai@ouhsc.edu (R.R.); courtney-houchen@ouhsc.edu (C.W.H.); parthasarathy-chandrakesan@ouhsc.edu (P.C.); 3 Department of Medicine, The University of Oklahoma Health Sciences Center, Oklahoma City, OK 73104, USA; 4 Department of Surgery, University of Oklahoma Health Sciences Center, Oklahoma City, OK 73104, USA; katherine-morris@ouhsc.edu; 5 VA Medical Center, Oklahoma City, OK 73104, USA

## Error in Figure

In the original publication [1], there was a mistake in Figures 5F and 6A as published. One of the images in Figure 4F was inadvertently duplicated in Figure 5F during our figure preparation process. A similar error was also noted between Figure 6A,D. The corrected Figure 5 and Figure 6 appear below. The authors state that the scientific conclusions are unaffected. This correction was approved by the Academic Editor. The original publication has also been updated.

## Figures and Tables

**Figure 5:**
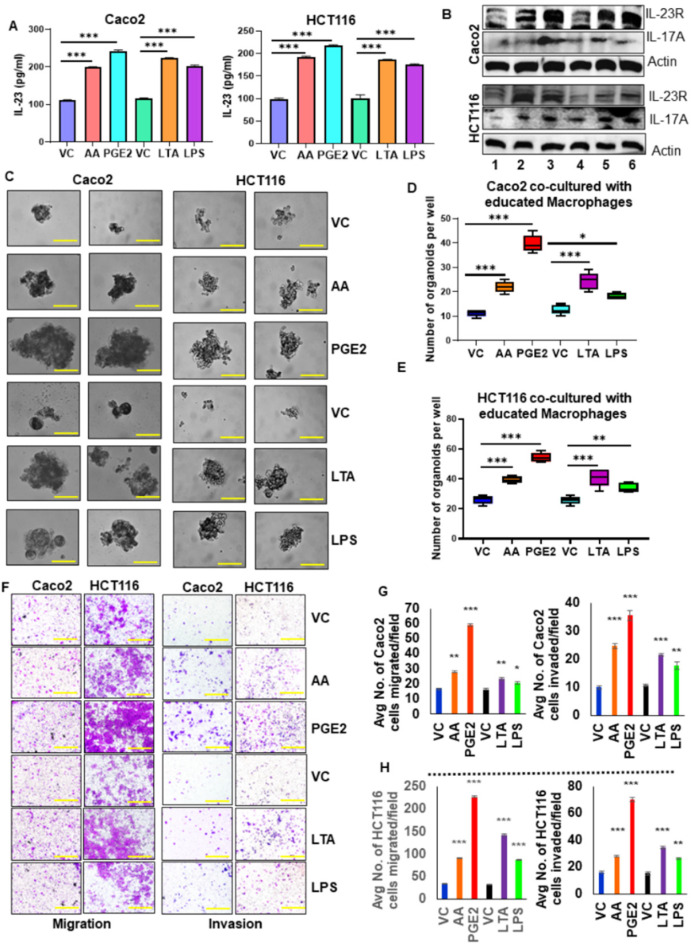
IL-23 production by macrophages enhances colon tumor cell aggressiveness. (**A**) The level of IL-23 in the spent media of the co-culture system (Caco2/HCT116 + educated macrophages with AA/PGE_2_/LTA/LPS) was measured using ELISA. (**B**) The expression of IL-23R, IL-17A were analyzed in Caco2 and HCT116 cells co-cultured with educated macrophages compared to uneducated macrophages. Lane1-Vehicle control, Lane2-AA, Lane3- PGE_2_, Lane4- Vehicle control, Lane5- LTA, Lane6- LPS. β-actin was used as a protein loading control. (**C**) Co-culture of educated macrophages with tumor cells increased the self-renewal ability of cancer cells compared with uneducated macrophages co-culture system (Magnification 40×). (**D**,**E**) Quantification of organoids formed by tumor cells co-cultured with educated macrophages compared to uneducated macrophages. (**F**) Migration and invasion assay showed that tumor cells co-cultured with educated macrophages increased migration and invasion compared to uneducated macrophages (Magnification 10×). (**G**,**H**) Quantification of the number of migrated and invaded cells. All experiments were performed a minimum of three times. Bars denote standard deviation (SD). * *p* < 0.05, ** *p* < 0.01, and *** *p* < 0.001 were considered statistically significant.

**Figure 6:**
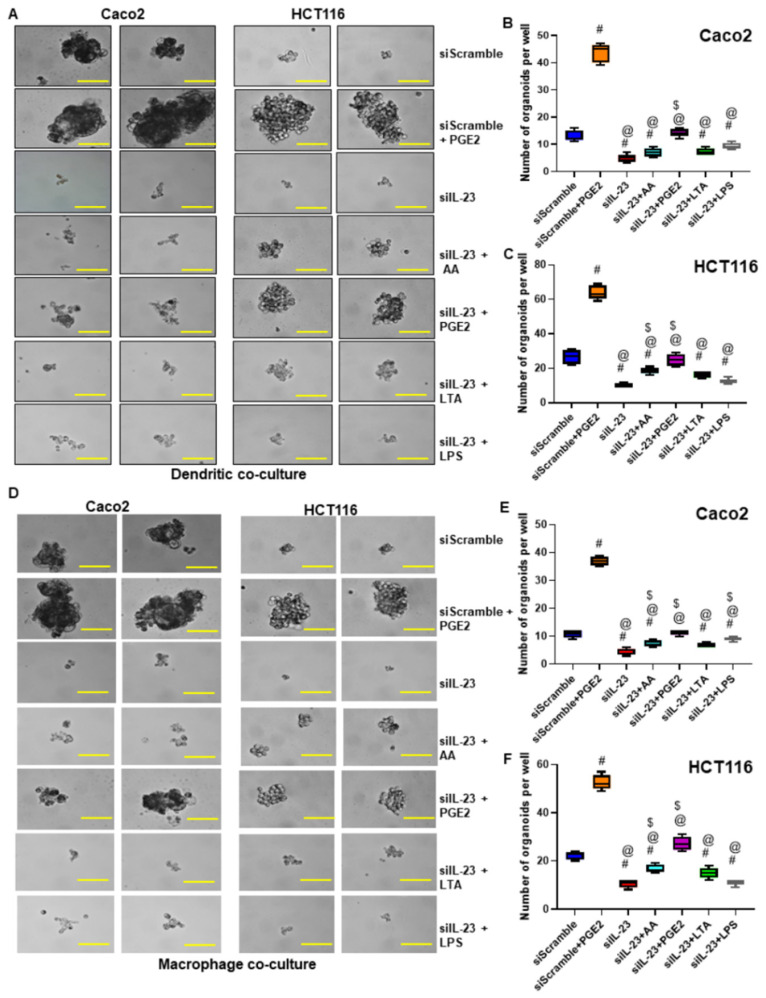
Inhibition of IL-23 in DCs and macrophages reduced colon tumor cell self-renewal. (**A**,**D**) Effect of siRNA knockdown of IL-23 in educated DCs and macrophages on the self-renewal ability of co-cultured Caco2 and HCT116 cells compared to scramble siRNA and scramble siRNA + PGE_2_ stimulated immune cells (Magnification 40×). (**B**,**C**,**E**,**F**) Quantification of organoids formed per well by tumor cells co-cultured with siIL-23 treated and educated DCs and macrophages compared to scramble siRNA treated and uneducated macrophages. #-compared with siScramble; @-compared with siScramble + PGE_2_; $-compared with siIL-23. All experiments were performed a minimum of three times. Bars denote standard deviation (SD).
