# Supplementary material for: Inflammatory Mediators and Gut Microbial Toxins Drive Colon Tumorigenesis by IL-23 Dependent Mechanism
Source: Cancers (Basel). 2021 Oct 14;13(20):5159. doi: 10.3390/cancers13205159 (PMC8533859; doi:10.3390/cancers13205159)
Supplement: Supplementary file 1 [file cancers-13-05159-s001.zip › cancers-1375103-supplementary.pdf]

Supplementary Materials

Inflammatory mediators and gut microbial toxins drive colon tumorigenesis by IL-23 dependent mechanism

Janani Panneerselvam, Venkateshwar Madka, Rajani Rai, Katherine T. Morris, Courtney W. Houchen, Parthasarathy Chandrakesan, Chinthalapally V. Rao

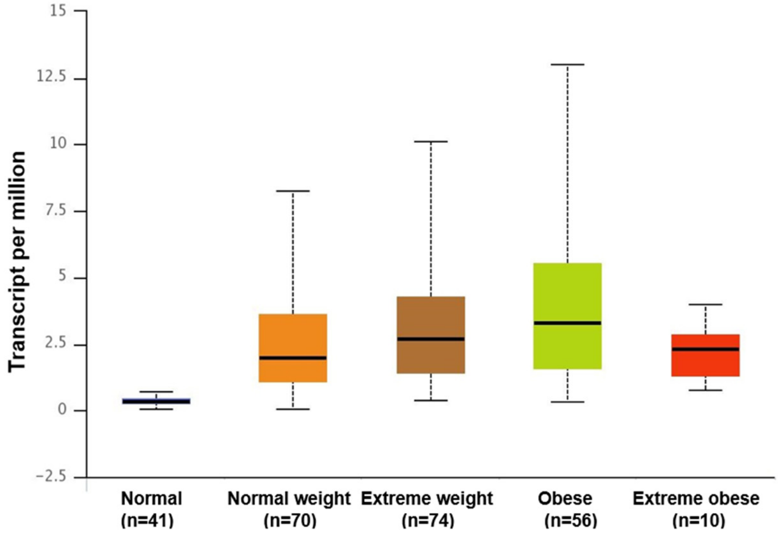

Comparison	Statistical significance
Normal-vs-Normal_Weight	1.82859999997564E-06
Normal-vs-Extreme_Weight	3.80050000026166E-07
Normal-vs-Obese	2.656100E-02
Normal-vs-Extreme_Obese	7.390500E-02
Normal_Weight-vs-Extreme_Weight	2.493800E-01
Normal_Weight-vs-Obese	2.598600E-01
Normal_Weight-vs-Extreme_Obese	6.560800E-01
Extreme_Weight-vs-Obese	4.730400E-01
Extreme_Weight-vs-Extreme_Obese	8.739200E-01
Obese-vs-Extreme_Obese	4.732400E-01

Figure S1A: The colon cancer samples downloaded from the TCGA database demonstrated the positive association between the expression of IL-23A and patient body weight.

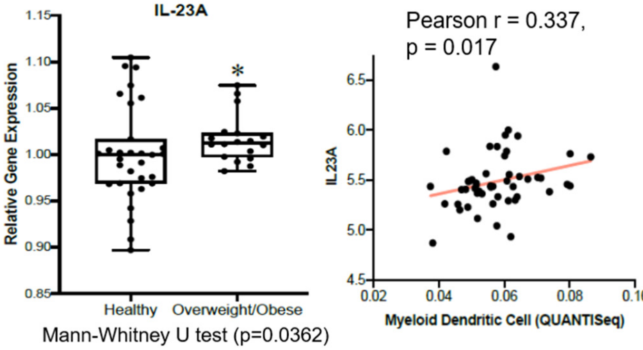

Figure S1B: All CRC gene microarray data was downloaded from the NCBI Gene Expression Omnibus. IL-23 expression was compared with healthy and overweight/obese patients and demonstrated the positive association between the expression of IL-23A and myeloid cell markers.

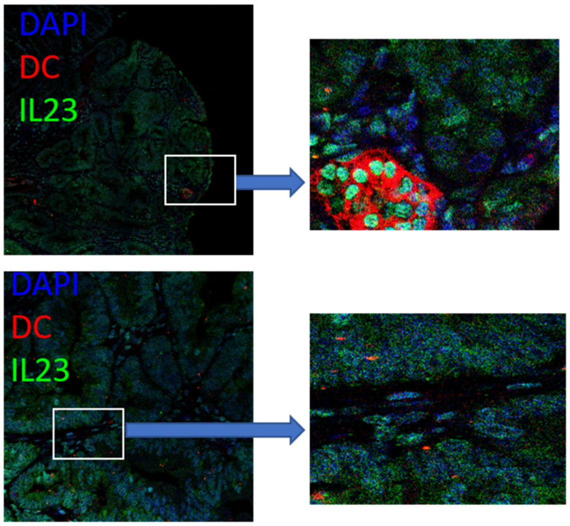

Figure S1C: IHC: staining of DC-sign co-stained with IL-23 in the rat colon cancer tissues.

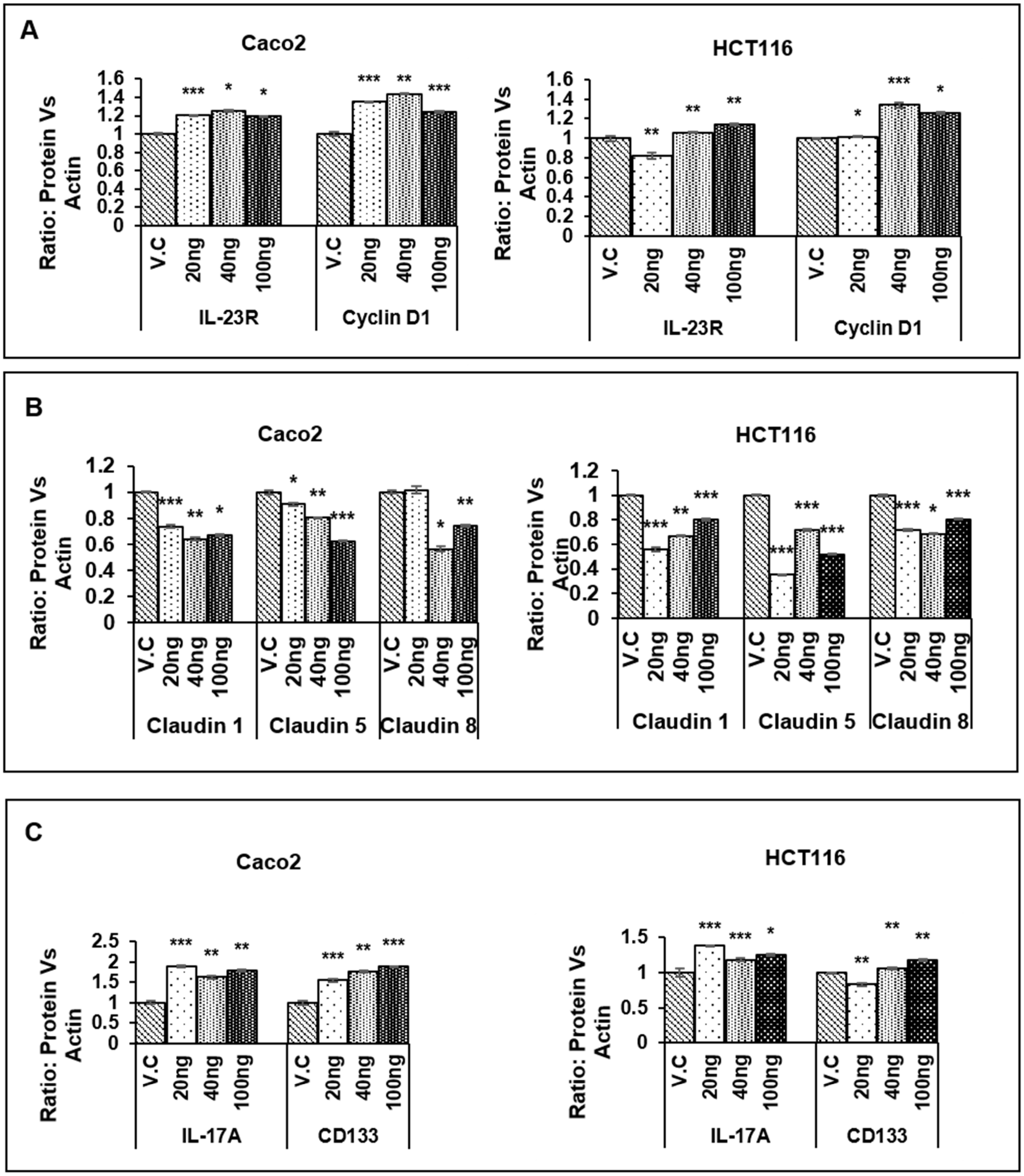

Figure S2A, S2B and S2C: Bar graph represent the semi-quantitative analysis of protein expression. Error bar denotes SD. $*p < 0.05$, $**p < 0.01$, and $***p < 0.001$.

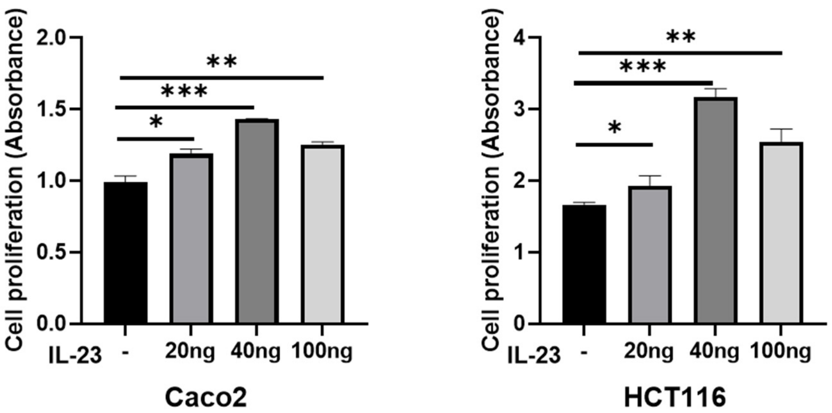

Figure S3A: Effect of rhIL-23 on cell proliferation of colon tumor cells. ∗p < 0.05, ∗∗p < 0.01, and ∗∗∗p < 0.001.

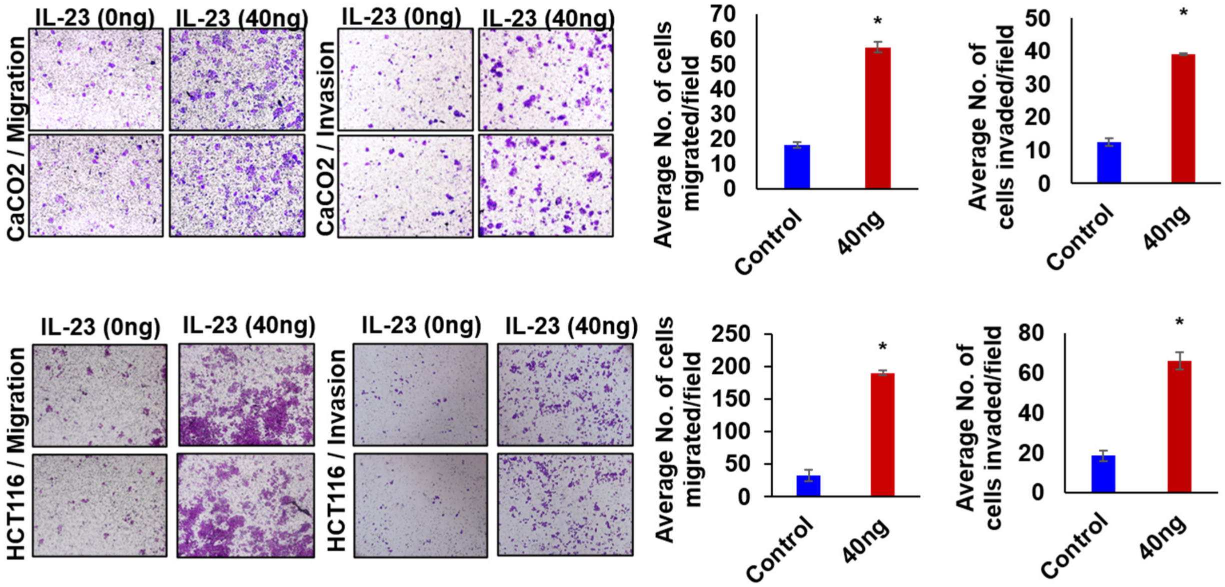

Figure S3B: Effect of rhIL-23 on migration and invasion of colon tumor cells. ∗p < 0.05, ∗∗p < 0.01, and ∗∗∗p < 0.001.

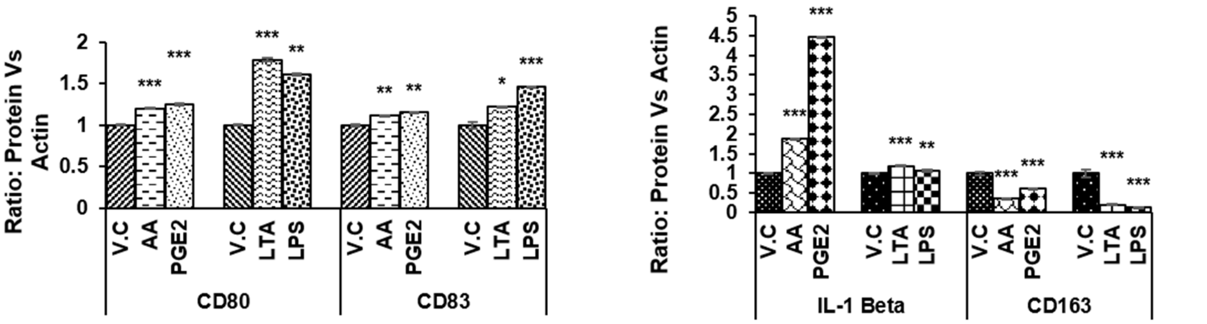

Figure S4A and S4B: Bar graph represent the semi-quantitative analysis of protein expression. Error bar denotes SD. ∗p < 0.05, ∗∗p < 0.01, and ∗∗∗p < 0.001.

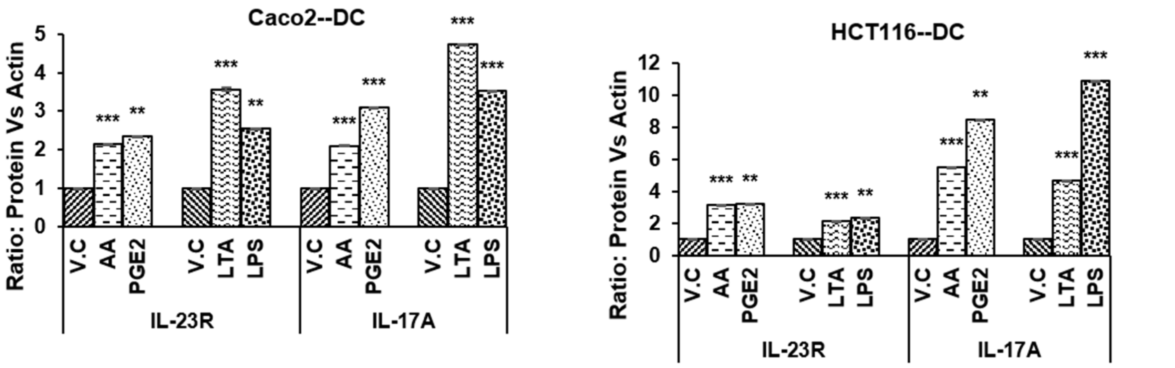

Figure S5A and S5B: Bar graph represent the semi-quantitative analysis of protein expression. Error bar denotes SD. ∗p < 0.05, ∗∗p < 0.01, and ∗∗∗p < 0.001.

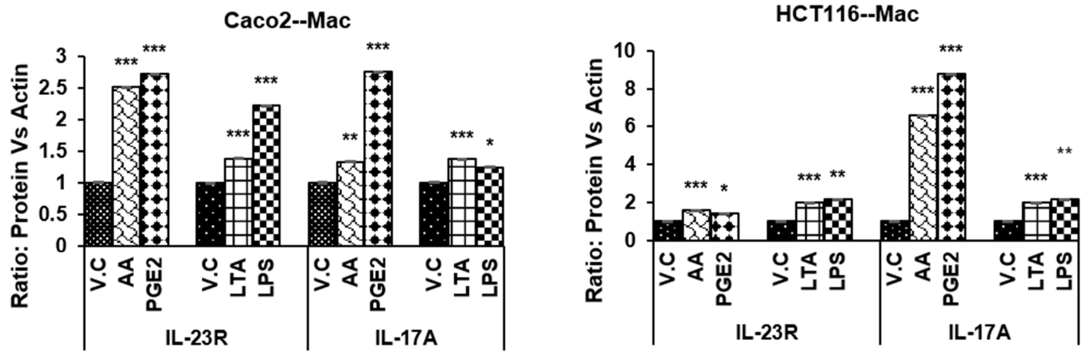

Figure S6A and B: Bar graph represent the semi-quantitative analysis of protein expression. Error bar denotes SD. $*p < 0.05$, $**p < 0.01$, and $***p < 0.001$.

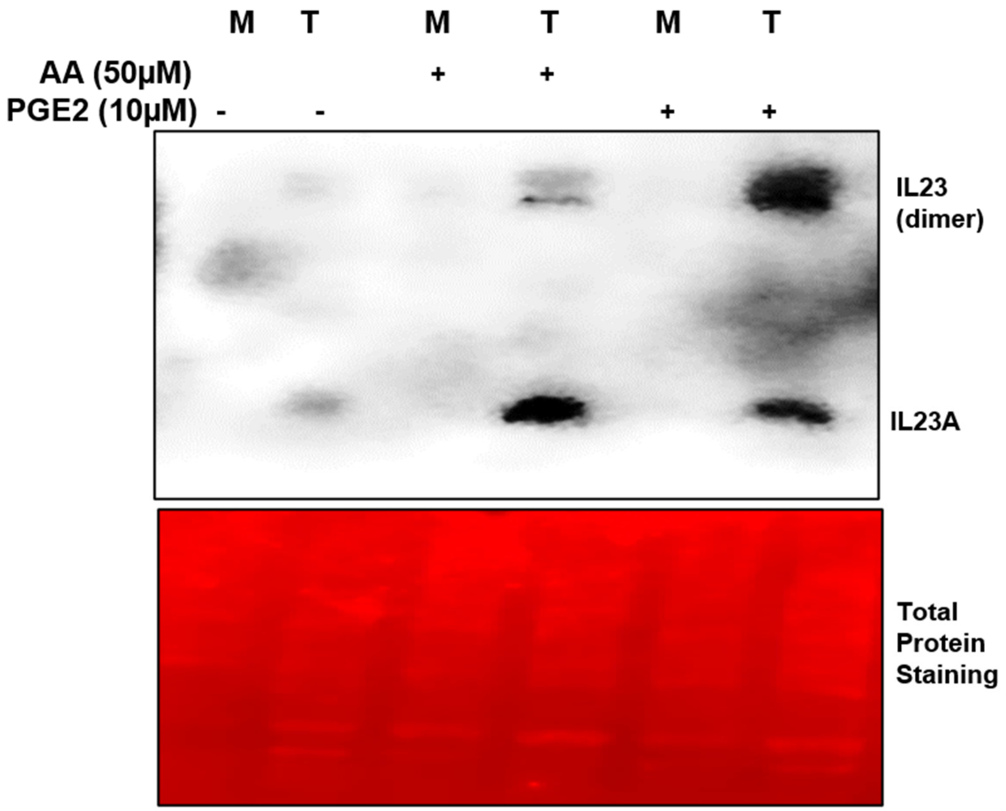

Figure S7: Ex vivo analysis of the effect of AA and PGE_2 on IL-23 expression in rat mucosa and colonic tumor.

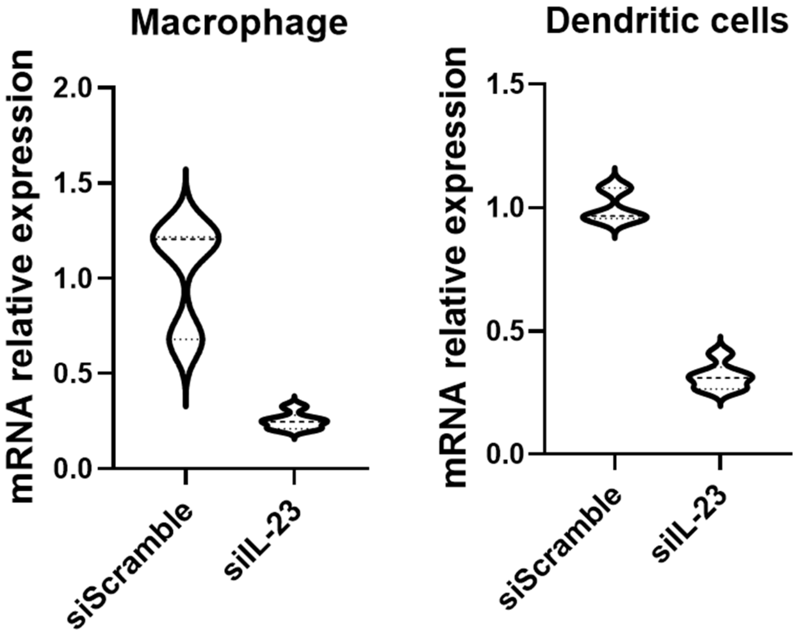

Figure S8 : THP-1 derived macrophages and dendritic cells were transfected with siIL-23 and knockdown of IL-23 expression was confirmed by RT-PCR analysis.

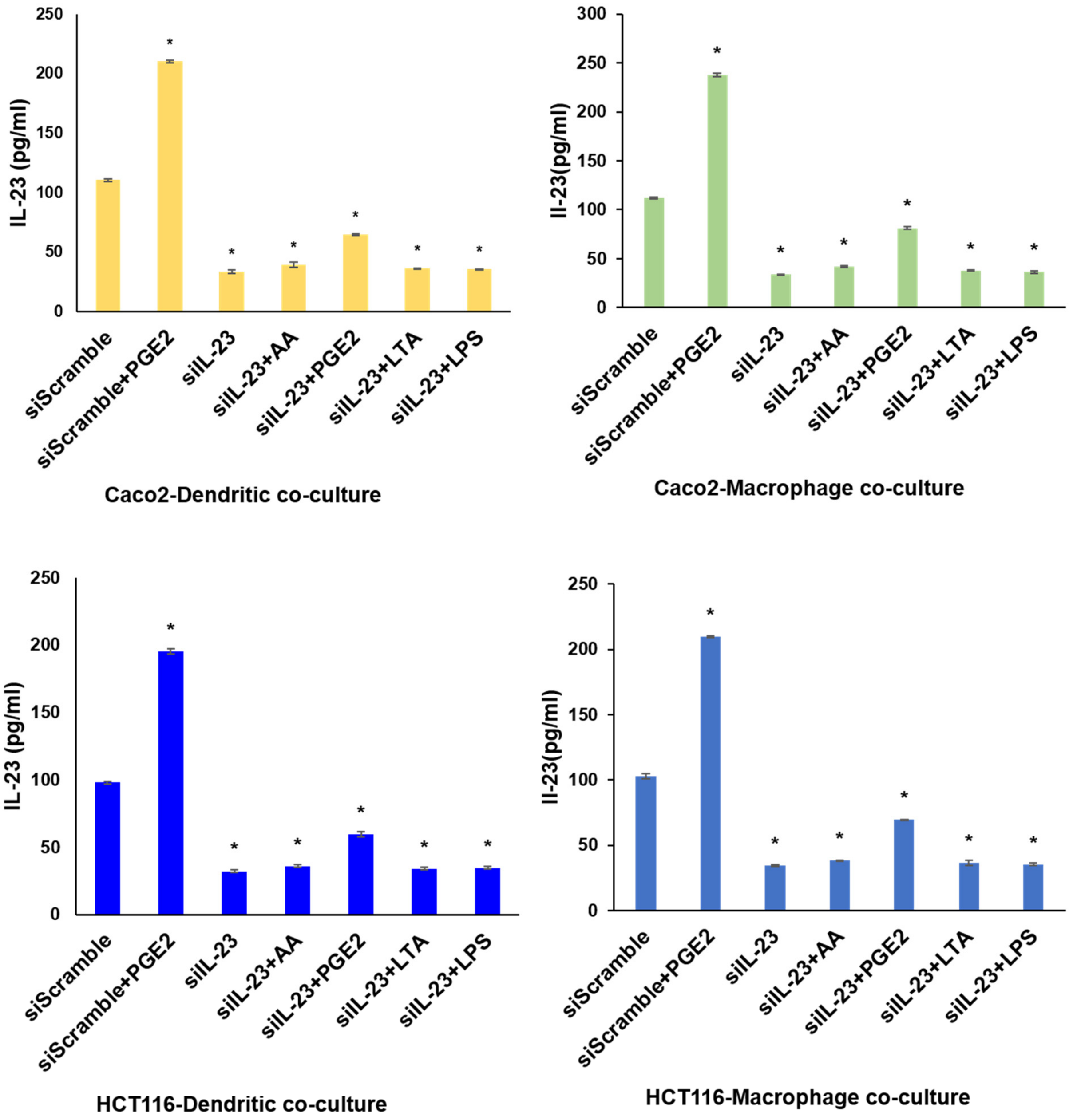

Figure S9: Effect of siRNA knockdown of IL-23 in educated DCs and macrophages on the level of IL-23 in the spent media of co-culture system (Caco2/HCT116+educated DCs/macrophages with AA/PGE$_2$/LTA/LPS) was measured using ELISA.

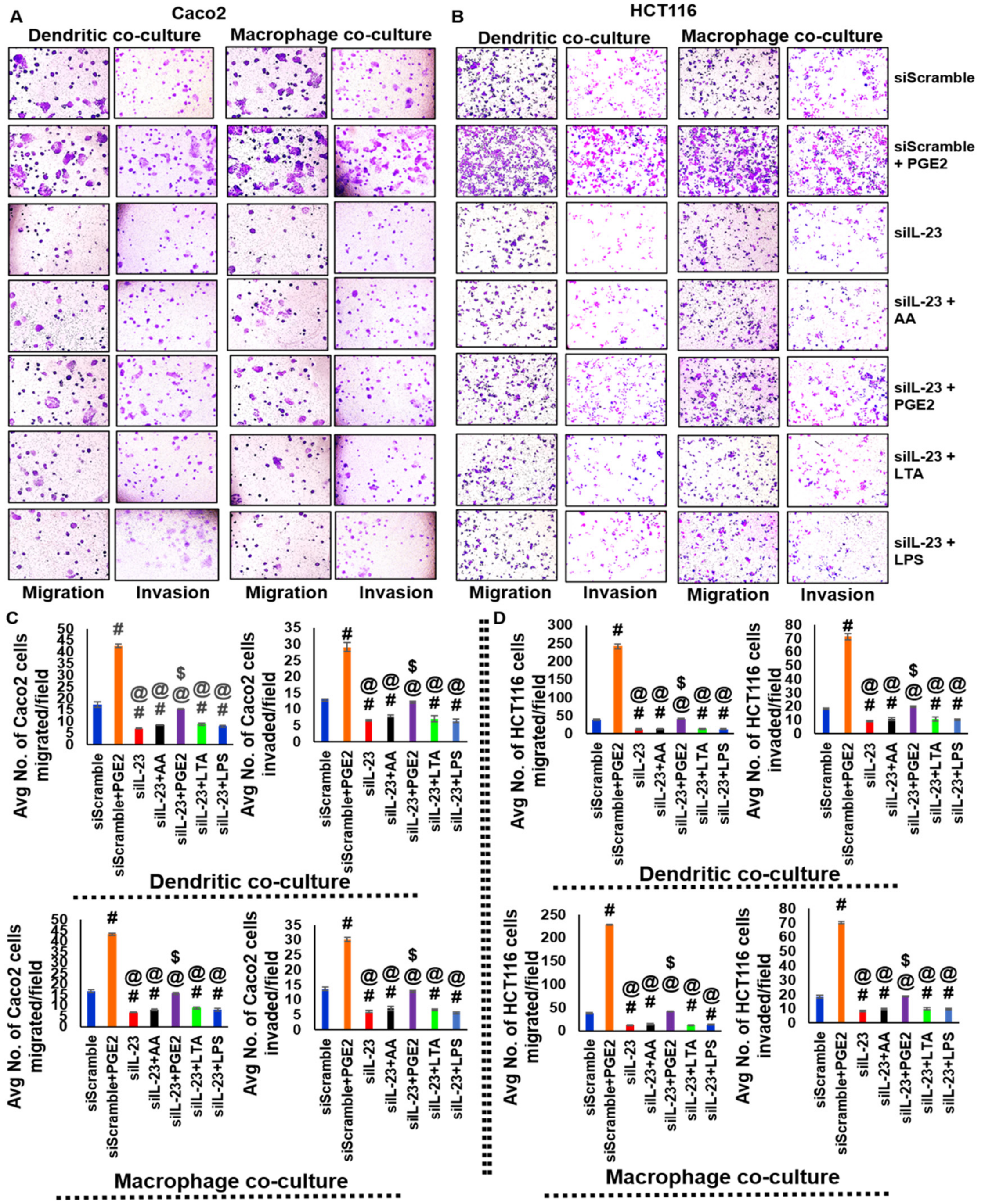

Figure S10: Inhibition of IL-23 in DCs and macrophages reduced colon tumor cell migration and invasion. (A, B) Effect of siRNA knockdown of IL-23 in educated DCs and macrophages on the cell migration and invasion ability of co-cultured Caco2 and HCT116 cells compared to scramble siRNA and scramble siRNA+PGE2 stimulated immune cells. (C, D) Quantification of the number of migrated and invaded tumor cells per field co-cultured with siIL-23 treated and educated DCs and macrophages compared to scramble siRNA treated and uneducated macrophages. # - compared with siScramble; @ - compared with siScramble+PGE2; $ - compared with siIL-23.

Figure S11: Original Blots

Figure 1E

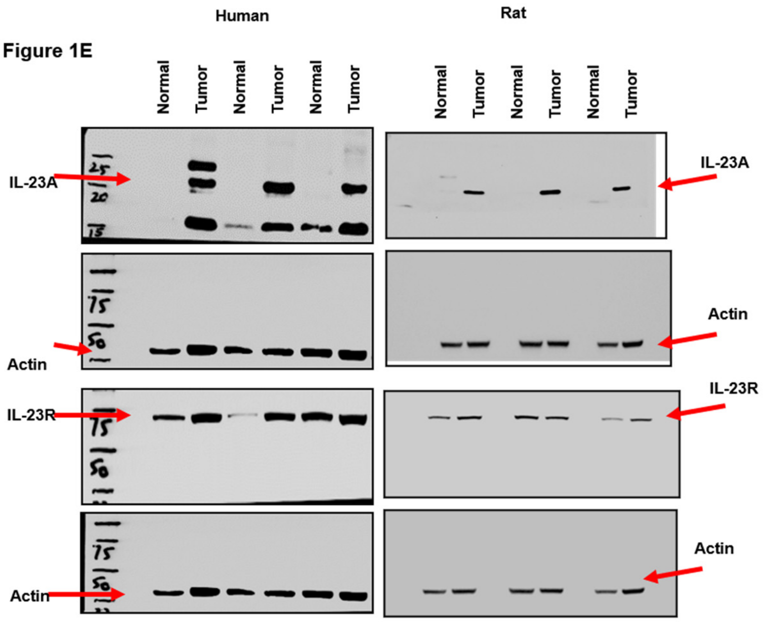

Figure 2A

Figure 2B

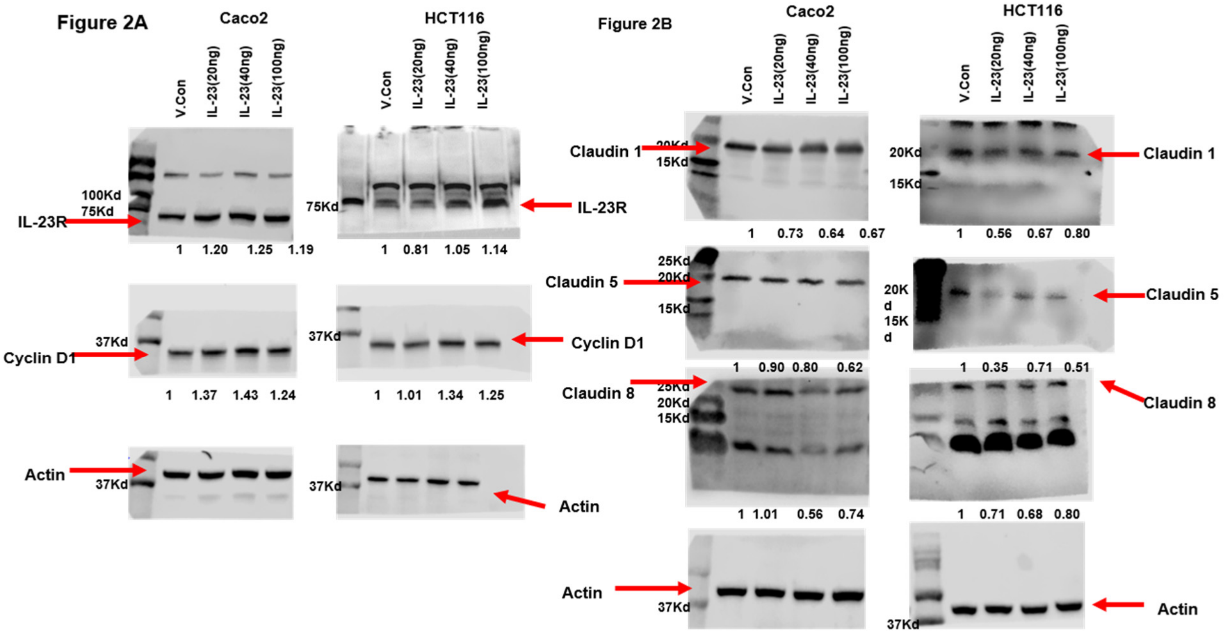

Figure S11: Original Blots

Figure 2C

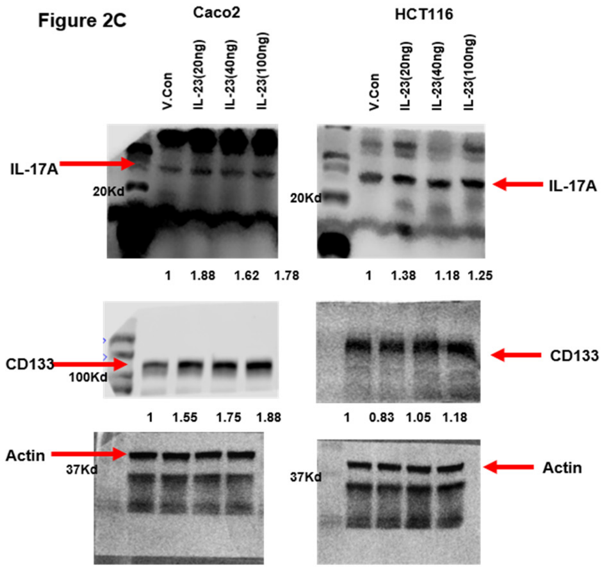

Figure 3D

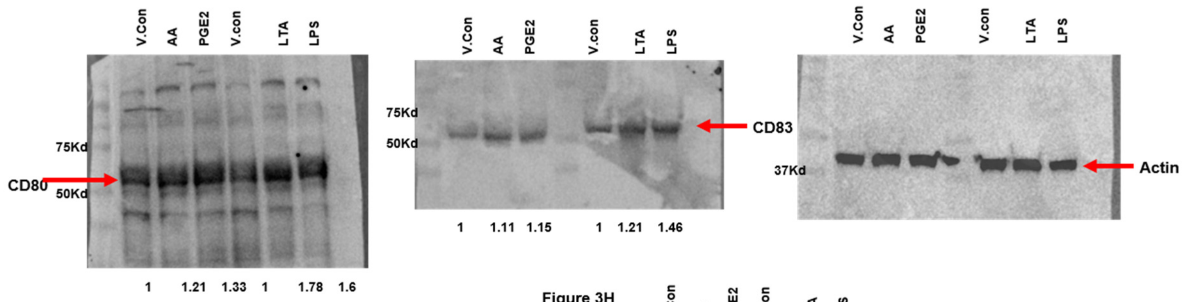

Figure 3E

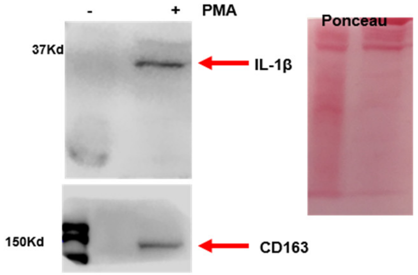

Figure 3H

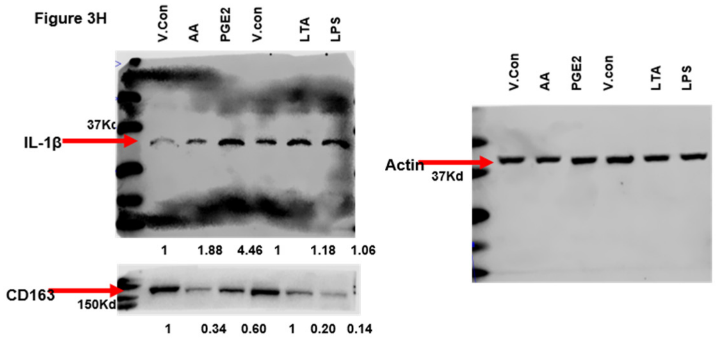

Figure S11: Original Blots

Figure 4B

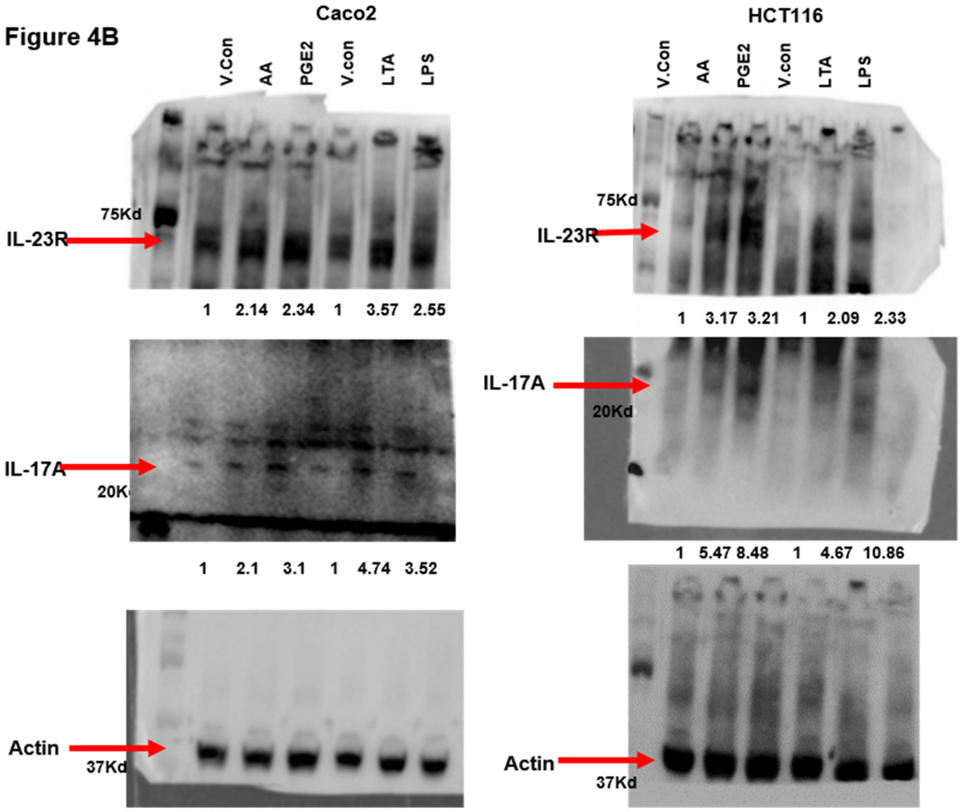

Figure 5B

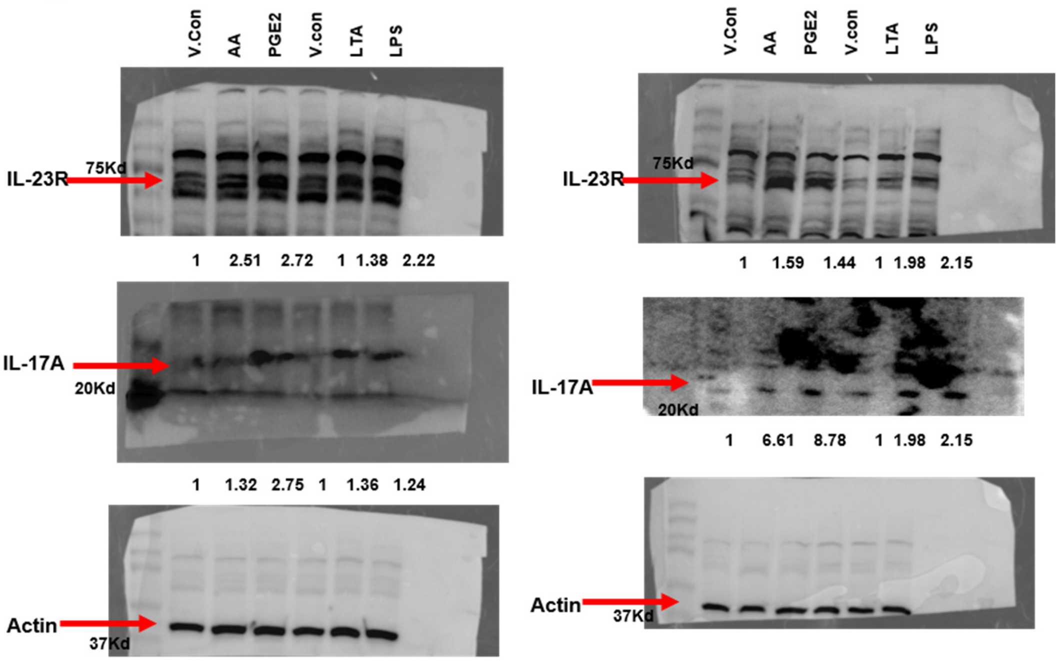

Reagents	Source
RPMI, DMEM, Penicillin/Streptomycin	Cat# MT10040CV, MT10013CV, 25-053-CI, Corning, Glendale, AZ, USA
FBS	Cat#S11550H, R and D systems, Minneapolis, MN, USA
rhIL-23	Cat#1290-IL, R and D Systems, Minneapolis, MN, USA
Phorbol-12-myristate-13-acetate, Arachidonic acid, PGE2	Cat#10008014, 90010,14010, Cayman Chemicals, Ann Arbor, MI, USA
rhGM-CSF, rhIL-4	Cat#130-095-372, 130-093-917, Miltenyi Biotec, Auburn, CA, USA
LTA	Cat#L2515-5MG, Sigma, St. Louis, MO, USA
LPS	Cat#sc-3535, Chemcruz, Dallas, TX, USA
MTT solution	Cat#30006, Biotium, CA, USA
Thinserts	Cat#662640, Greiner bio-one, Monroe, NC, USA
Matrigel coated trans-wells and transwells	Cat#08774122, 0877121, Fisher Scientific, Waltham, MA, USA
Human IL-23 ELISA kit	Cat#D2300B, R and D Systems, Minneapolis, MN, USA
Scramble siRNA and IL-23 siRNA	Cat#sc-37007, sc-43860, Santa Cruz Biotech, Dallas, TX, USA
Viromer blue transfection reagent	Cat#TT100300S, Origene, Rockville, MD, USA
Trizol	Cat#15596026, Invitrogen, Carlsbad, CA, USA
iScript cDNA synthesis kit	Cat#170-8891, Bio-Rad, Hercules, CA, USA
iQTM SYBR Green supermix	Cat#1706662, Bio-Rad, Hercules, CA, USA
hIL-23A- primers [Forward-CCCAGCAAGGCCAAGATAAA, Reverse-ACATATGCAGGTCCCACTAA] h18S- primers [Forward-GCTTAATTTGACTCAACACGGGA, Reverse-AGCTATCAATCTGTCAATCCTGTC]	Integrated DNA Technologies, Coralville, IA, USA
BCA protein assay	Cat#23225, Pierce, Rockford, IL, USA
ECL Western blotting detection reagents	Cat#32106, Thermo Scientific, Waltham, MA, USA
Slow fade Gold antifade reagent	Cat#S36937, Life technologies, Carlsbad, CA, USA
IL-23A (1:1000), IL-23R (1:1000), CyclinD1 (1:3000), Claudin 1 (1:1000), Claudin 5 (1:1000), CD133 (1:1000), CD80 (1:1000)	Cat#ab45420, ab175072, ab134175, ab15098, ab53765, ab19898, ab86473, Abcam, Cambridge, MA, USA
Claudin 8 (1:1000)	Cat#40-0700Z, Invitrogen, Carlsbad, CA, USA
IL-17A (1:1000)	Cat#A00421-2, Boster, Pleasanton, CA, USA
CD83 (1:1000), IL-1Beta (1:1000), CD163 (1:1000), DC-sign (1:400), Beta Actin (1:2000)	Cat#99075, 12703, 93498, 13193, 4970, Cell Signaling Technology, Danvers, MA, USA
Alexa Fluor® 488 conjugated Ab (1:500)	Cat#A11034, Life Technologies, Carlsbad, CA, USA
DAPI	Cat#D21490, Invitrogen, Carlsbad, CA, USA
